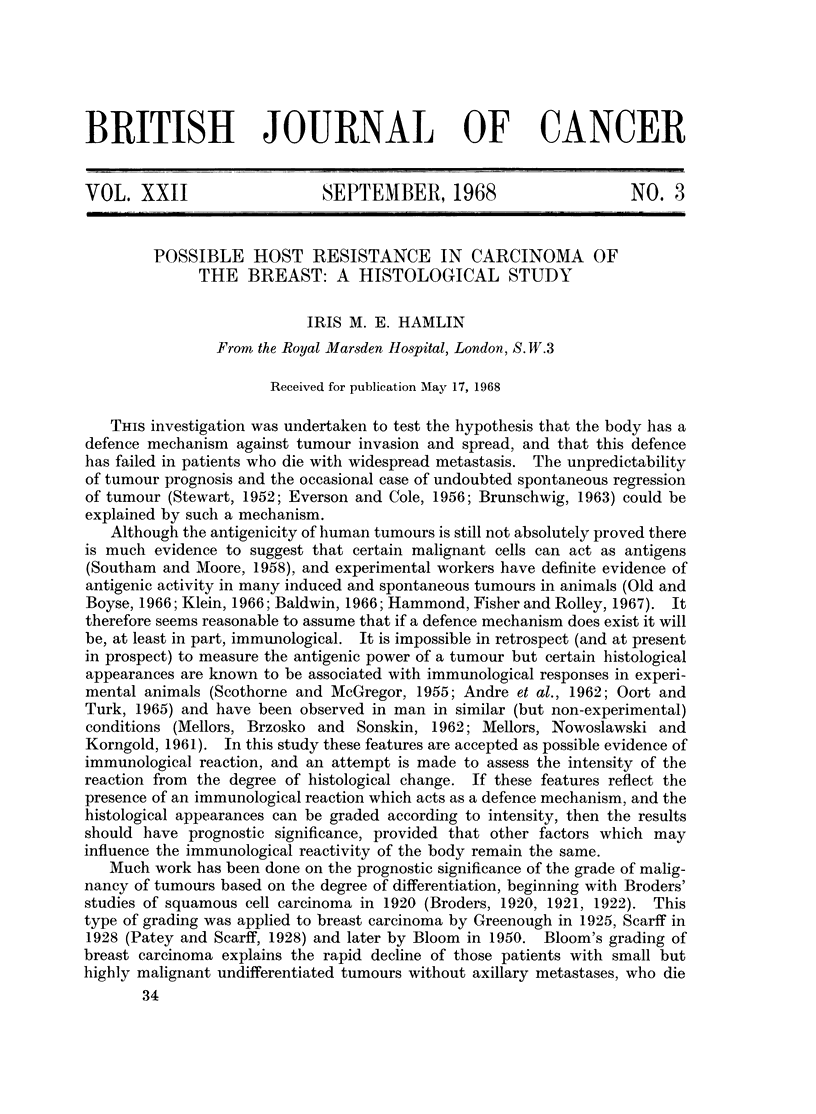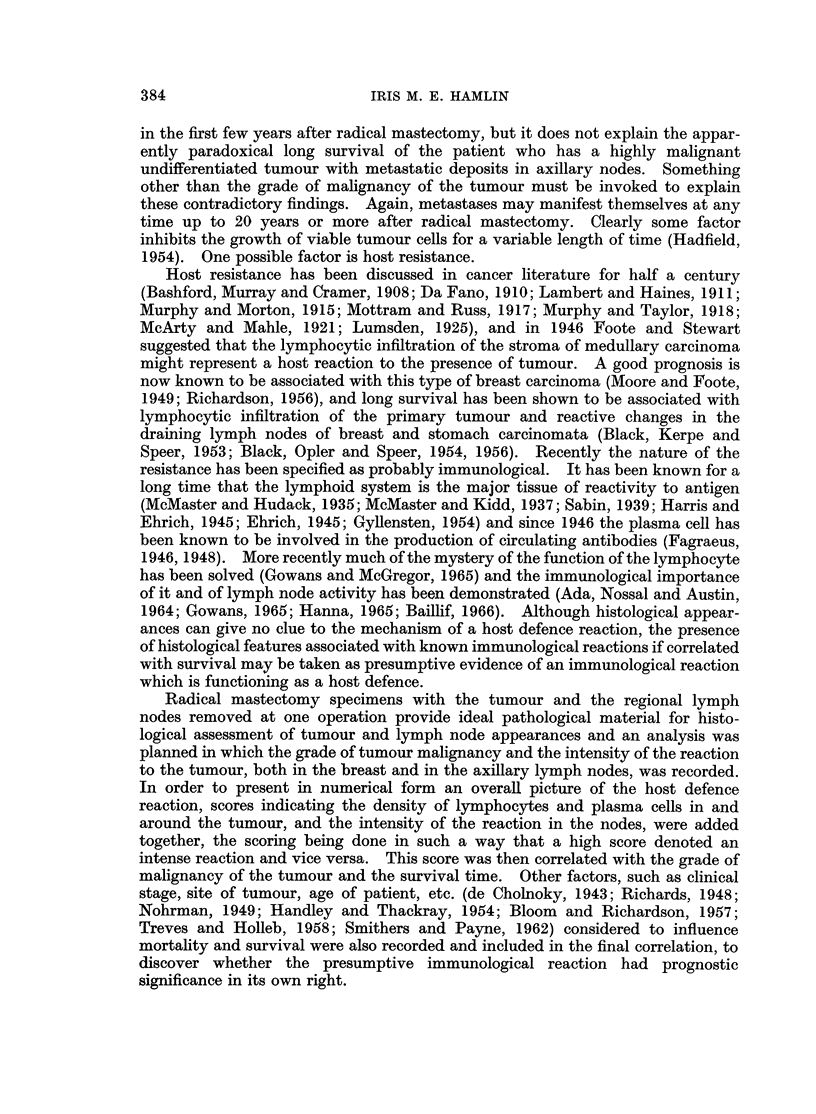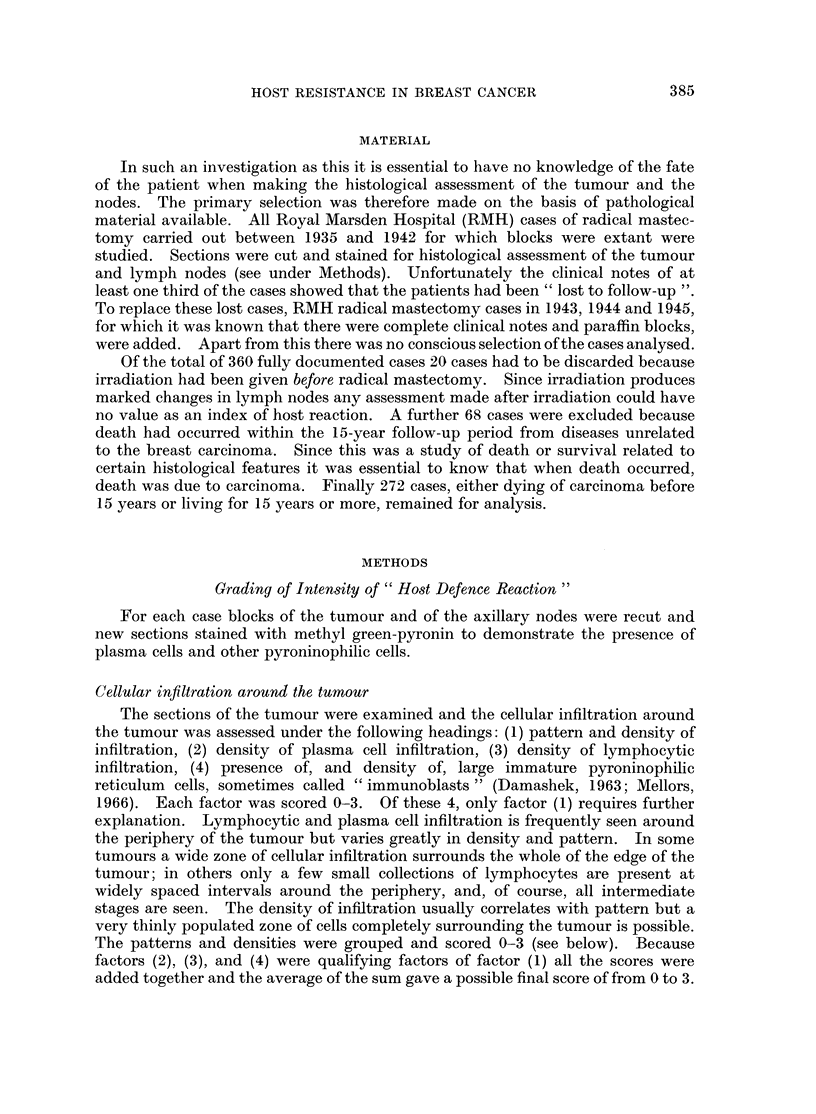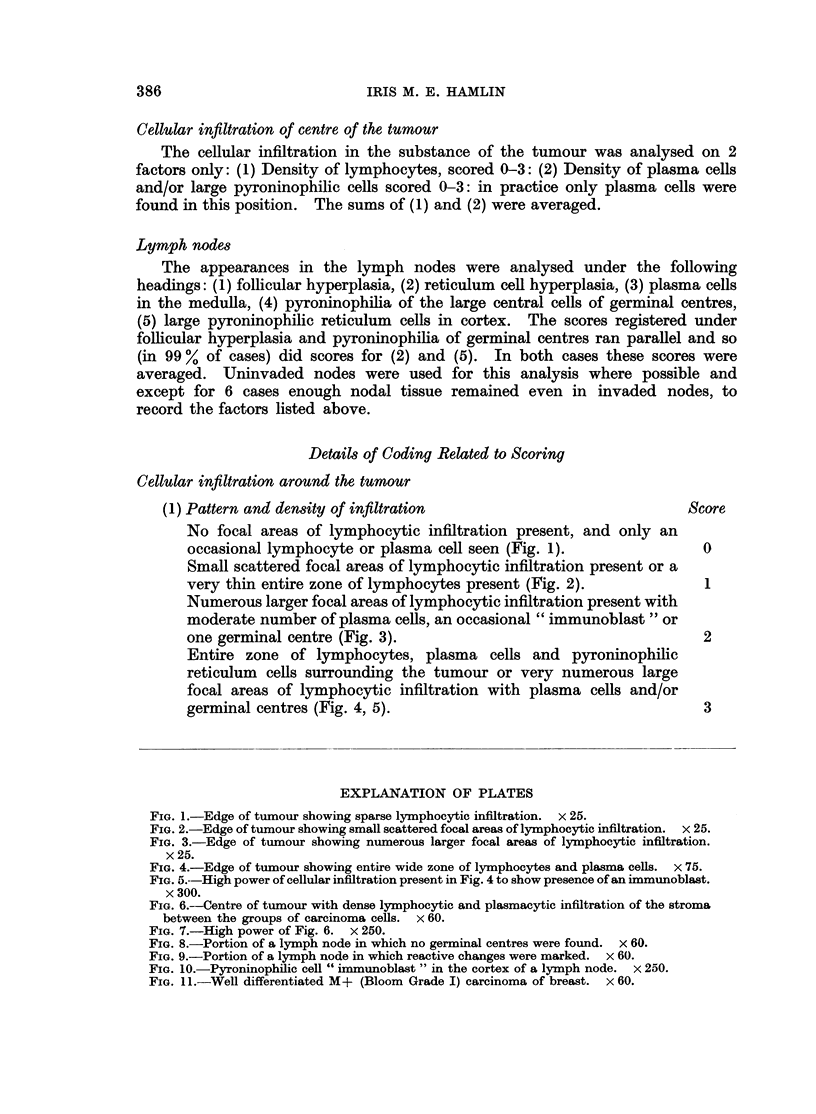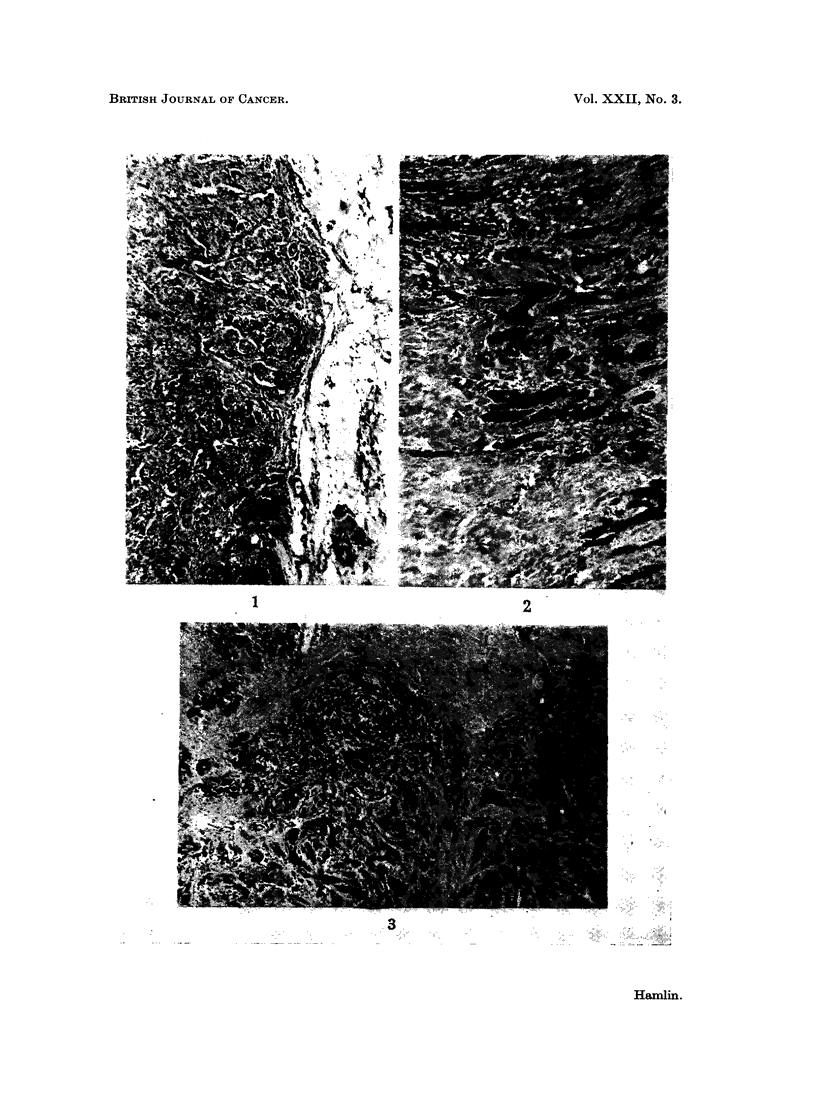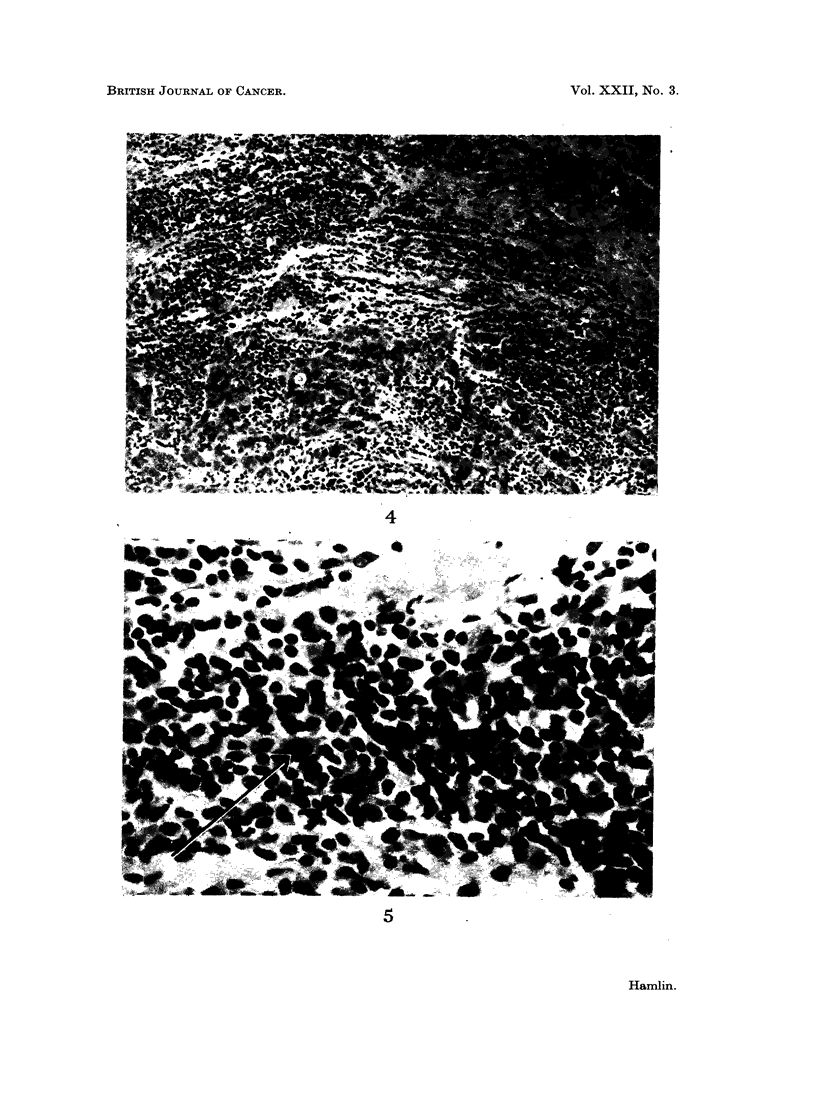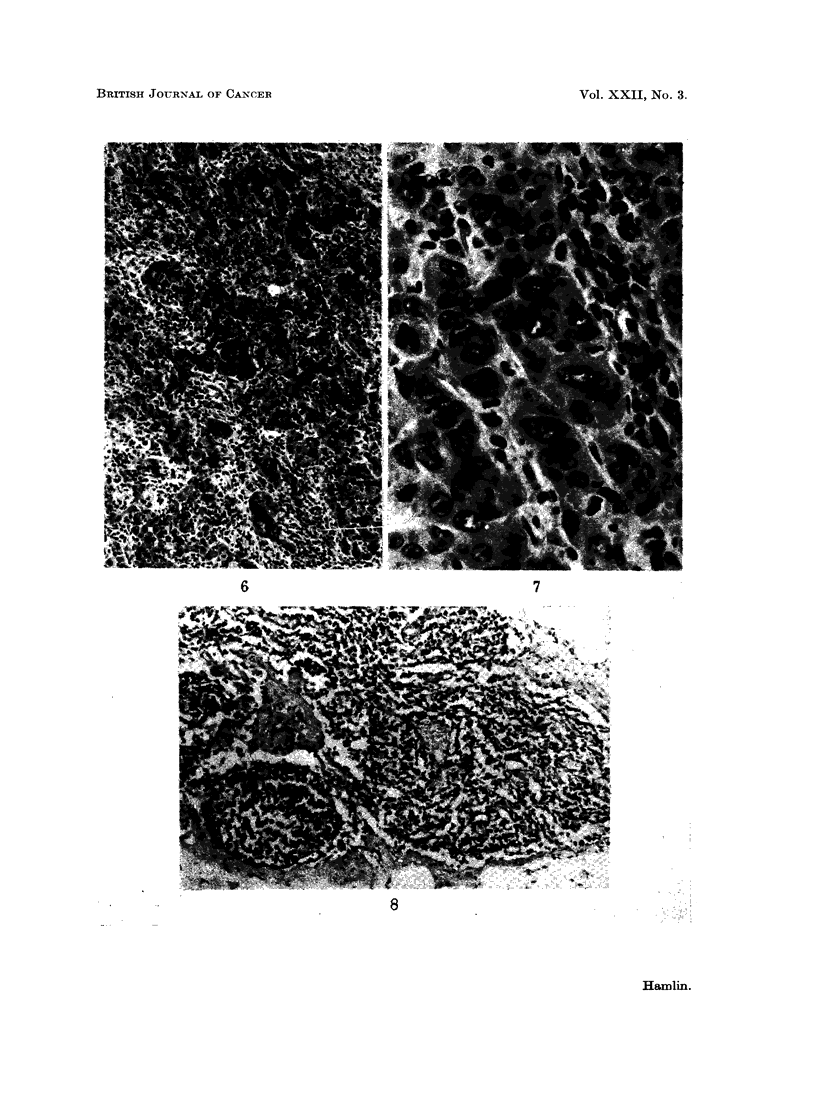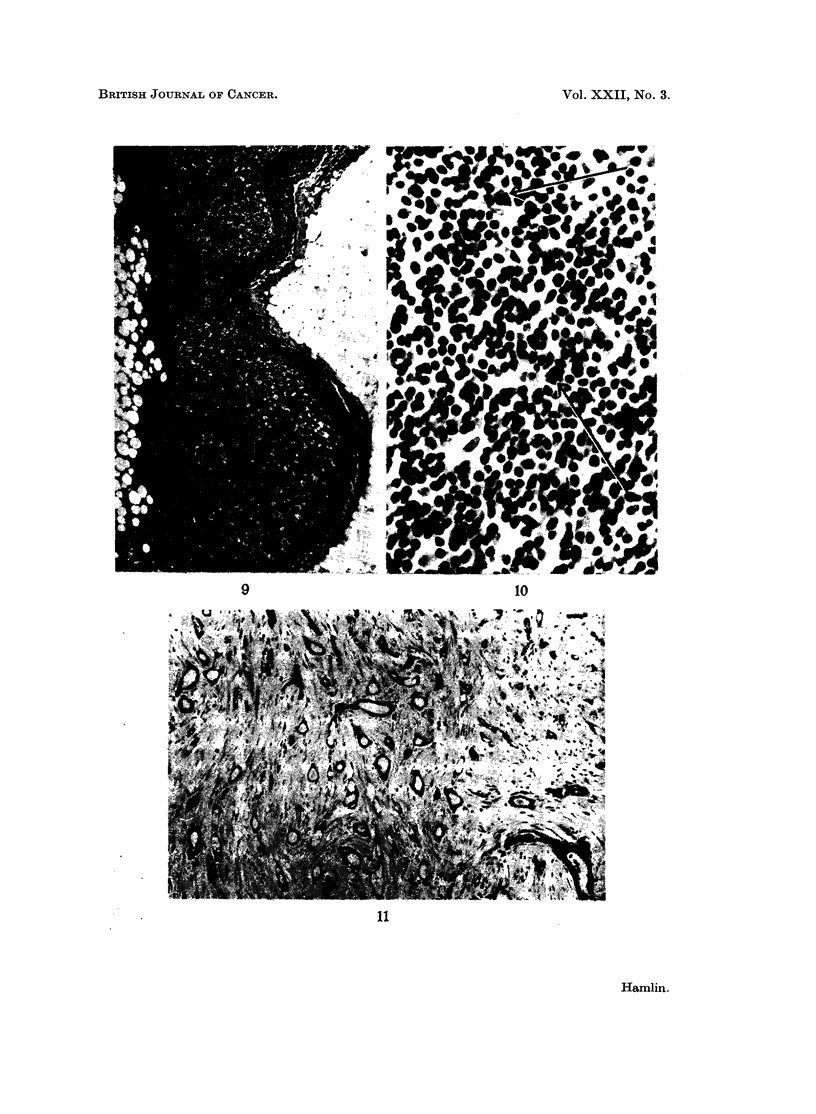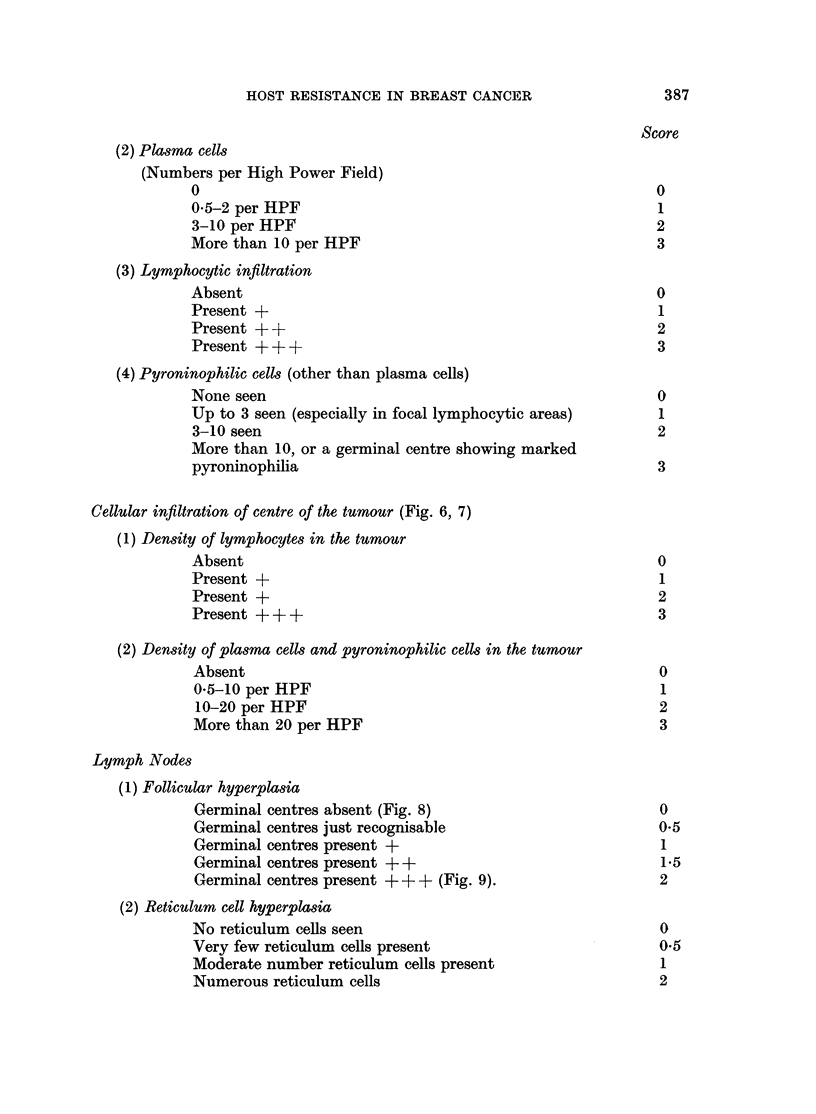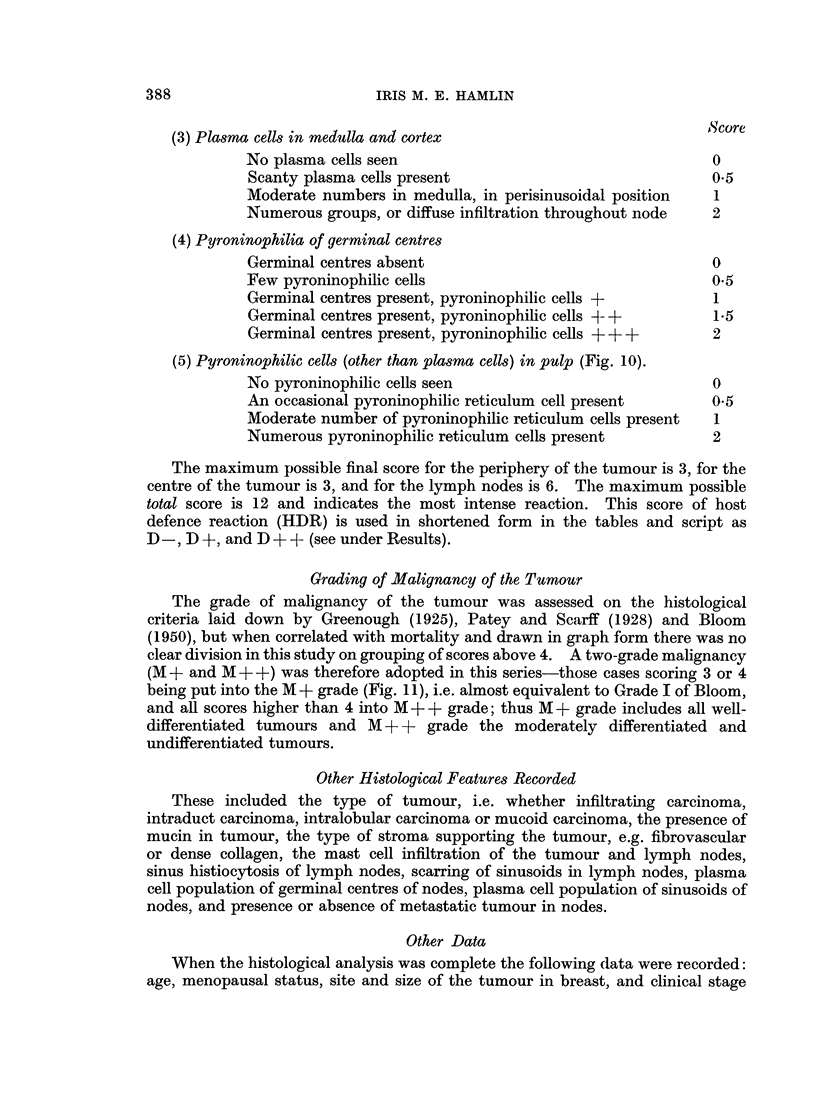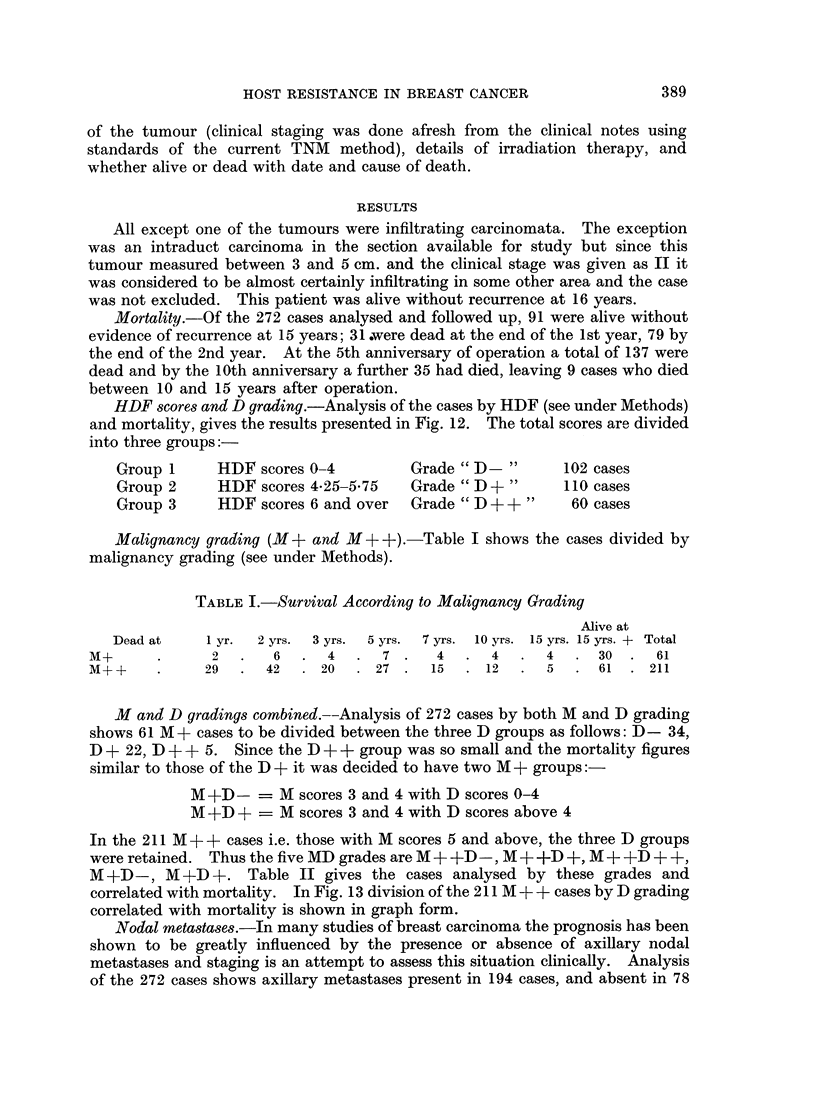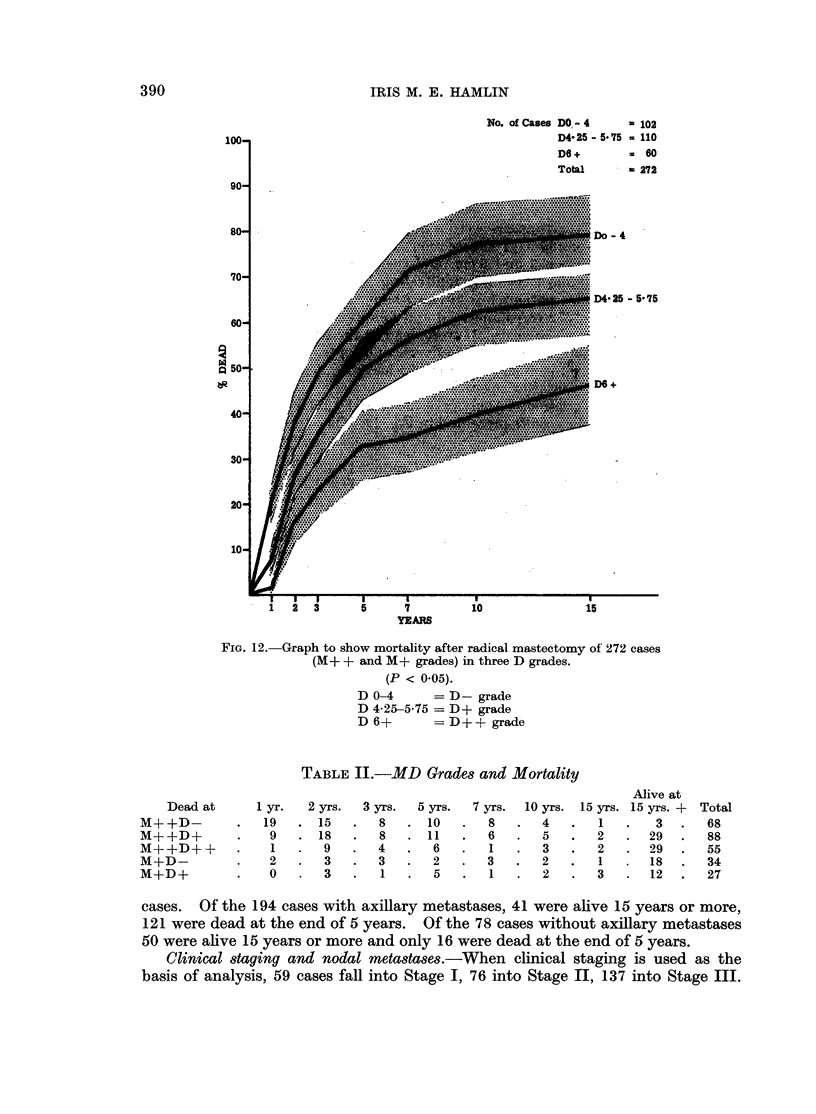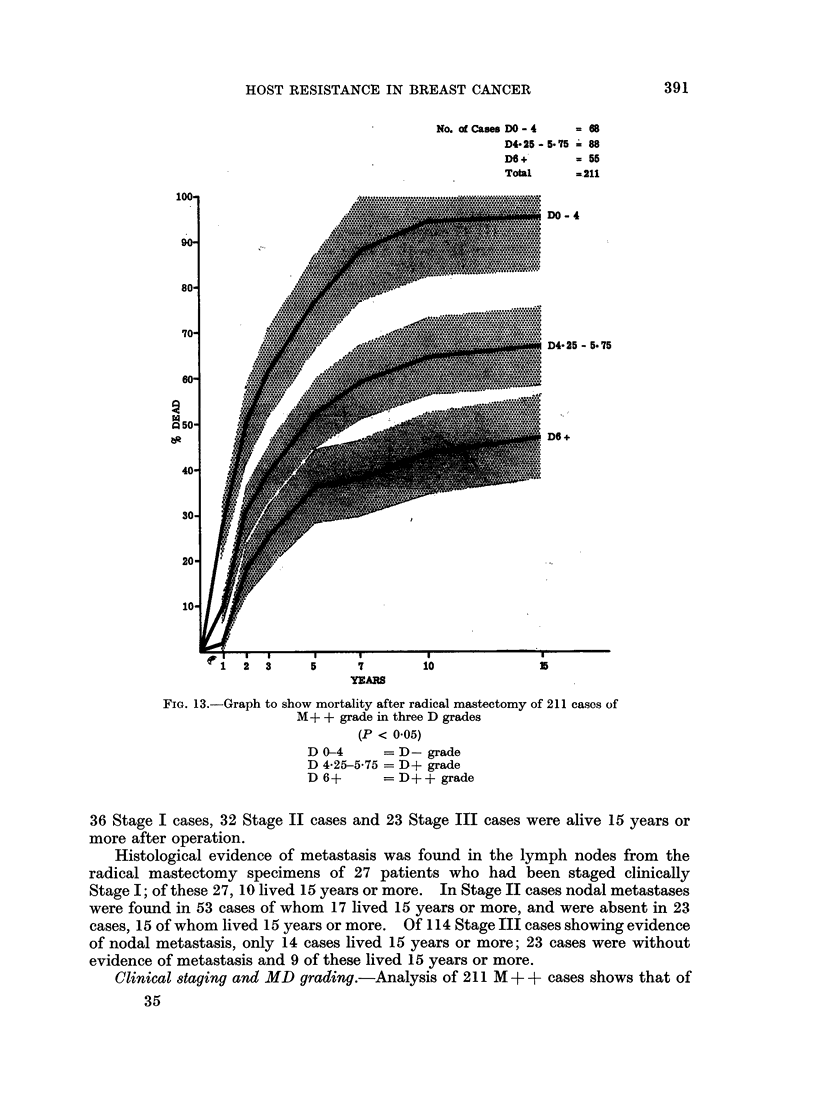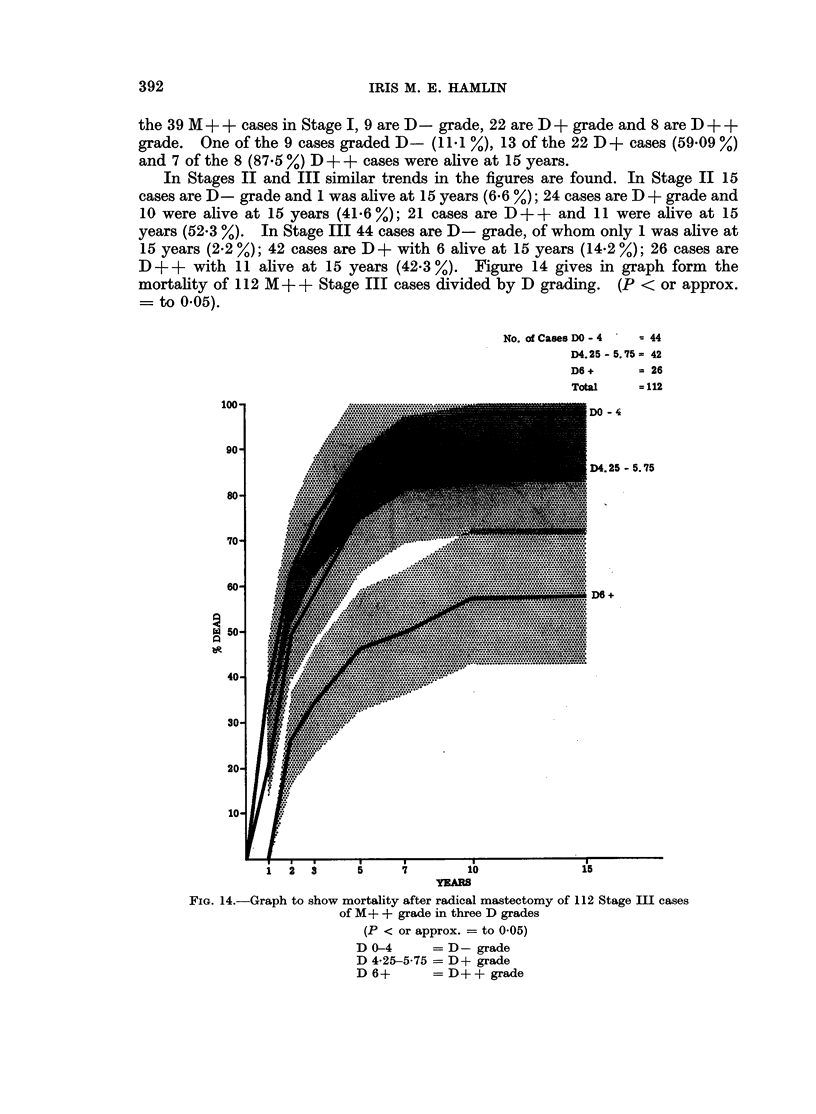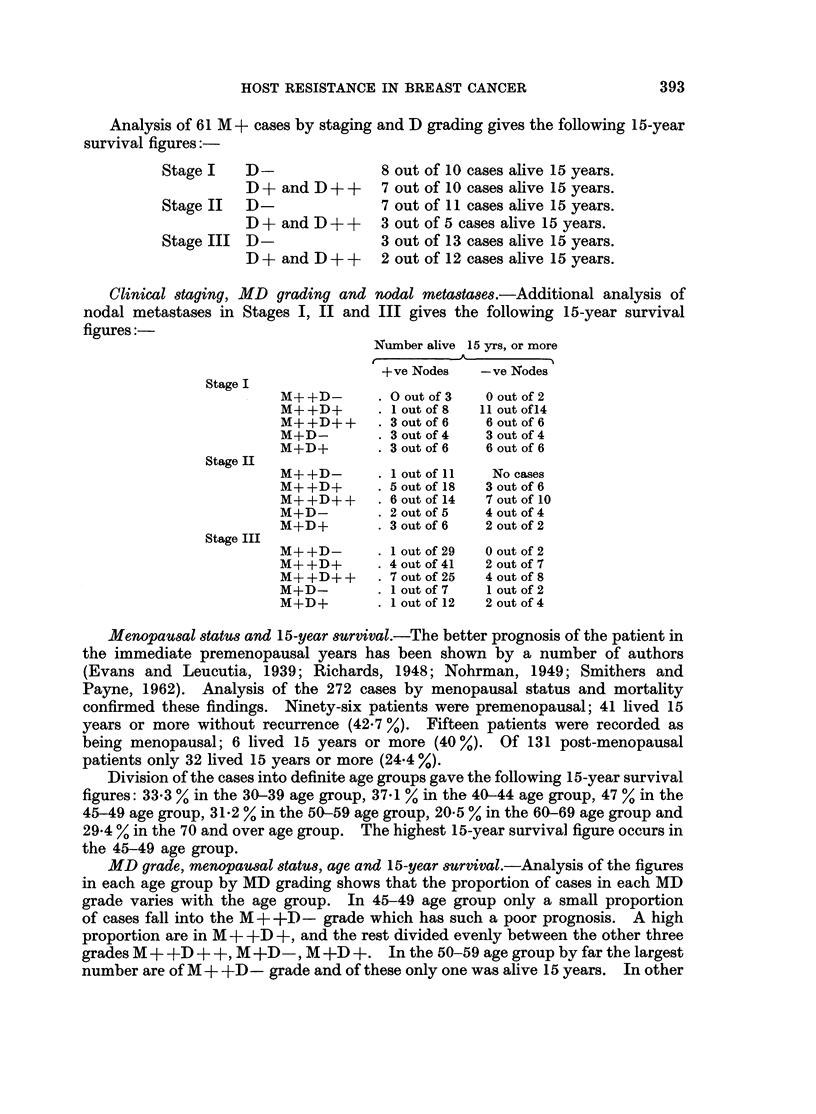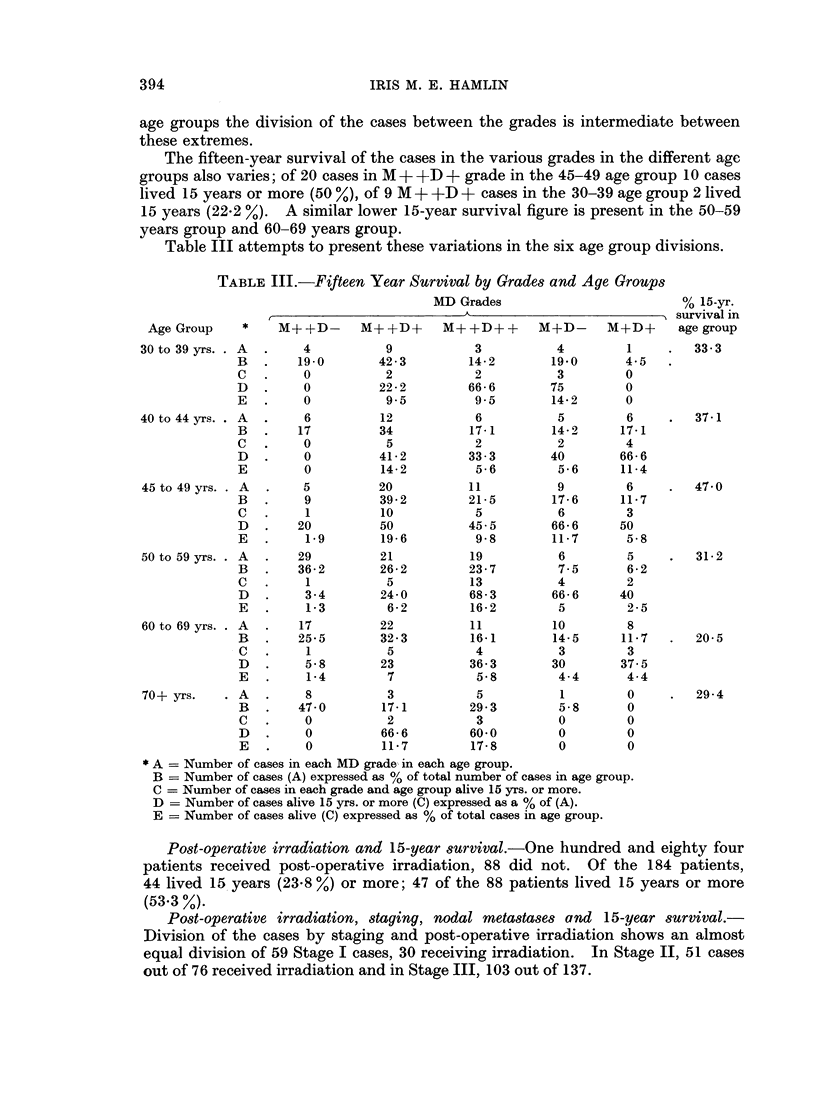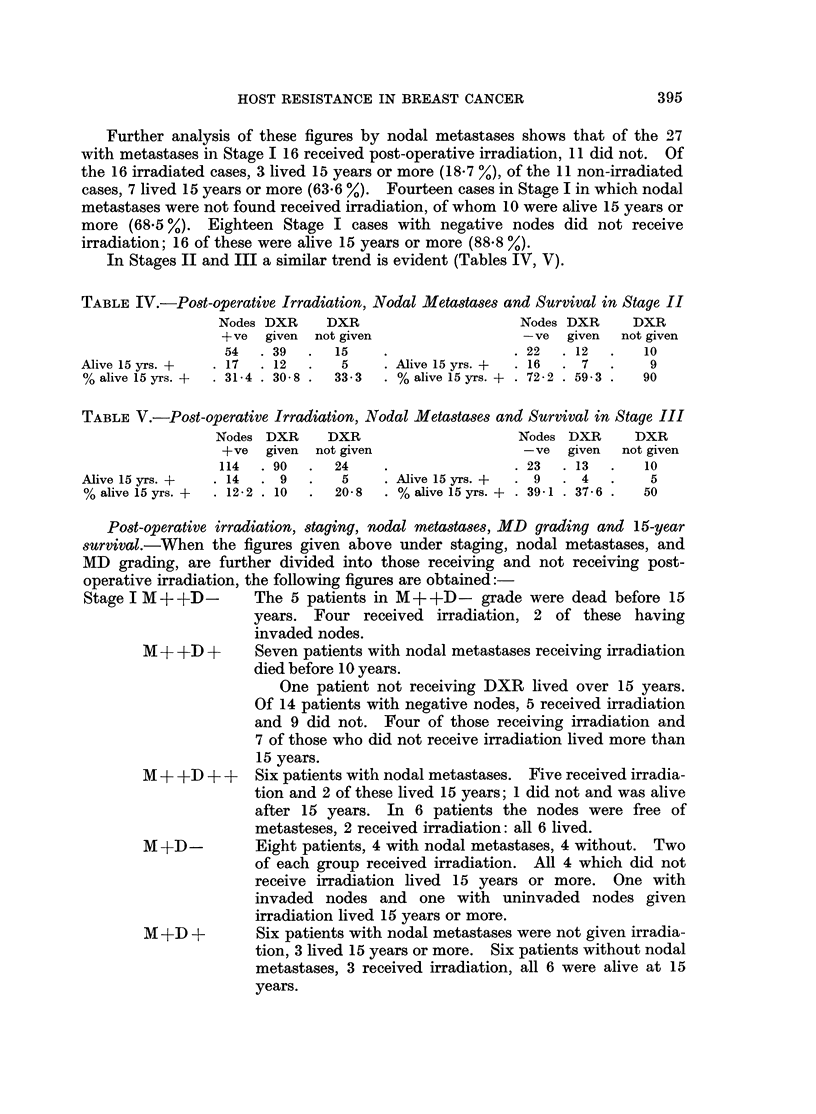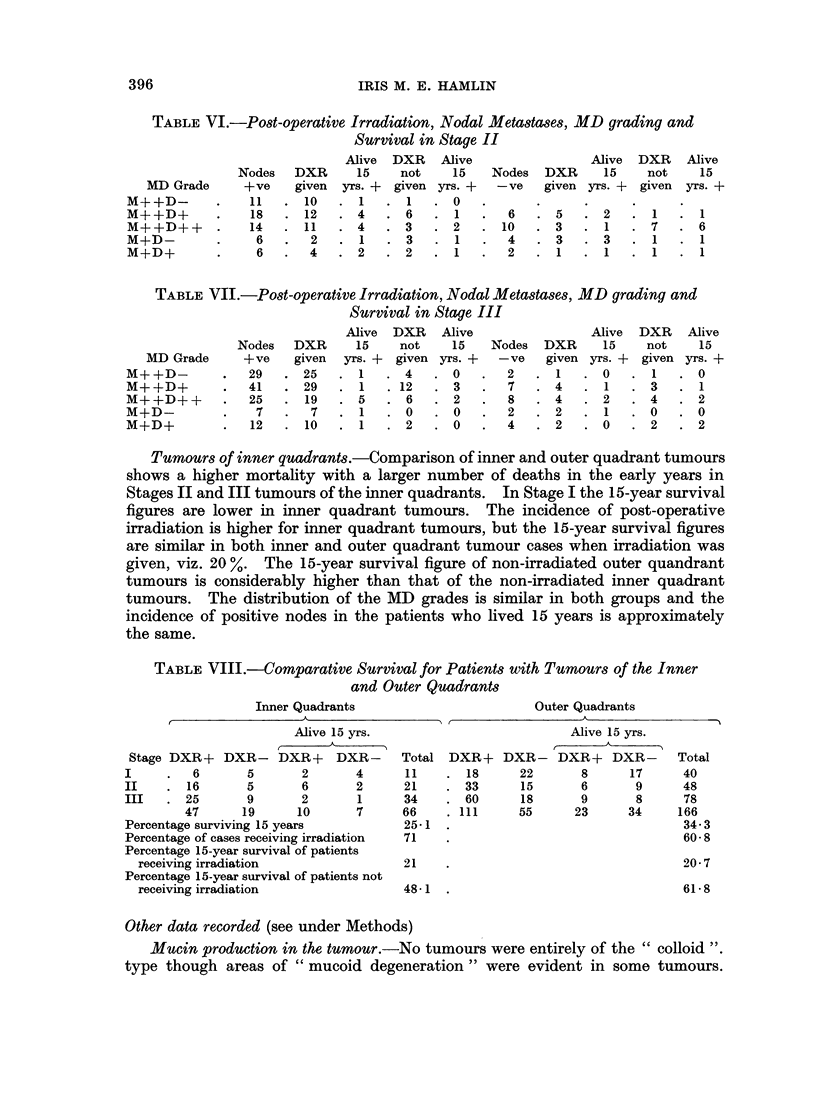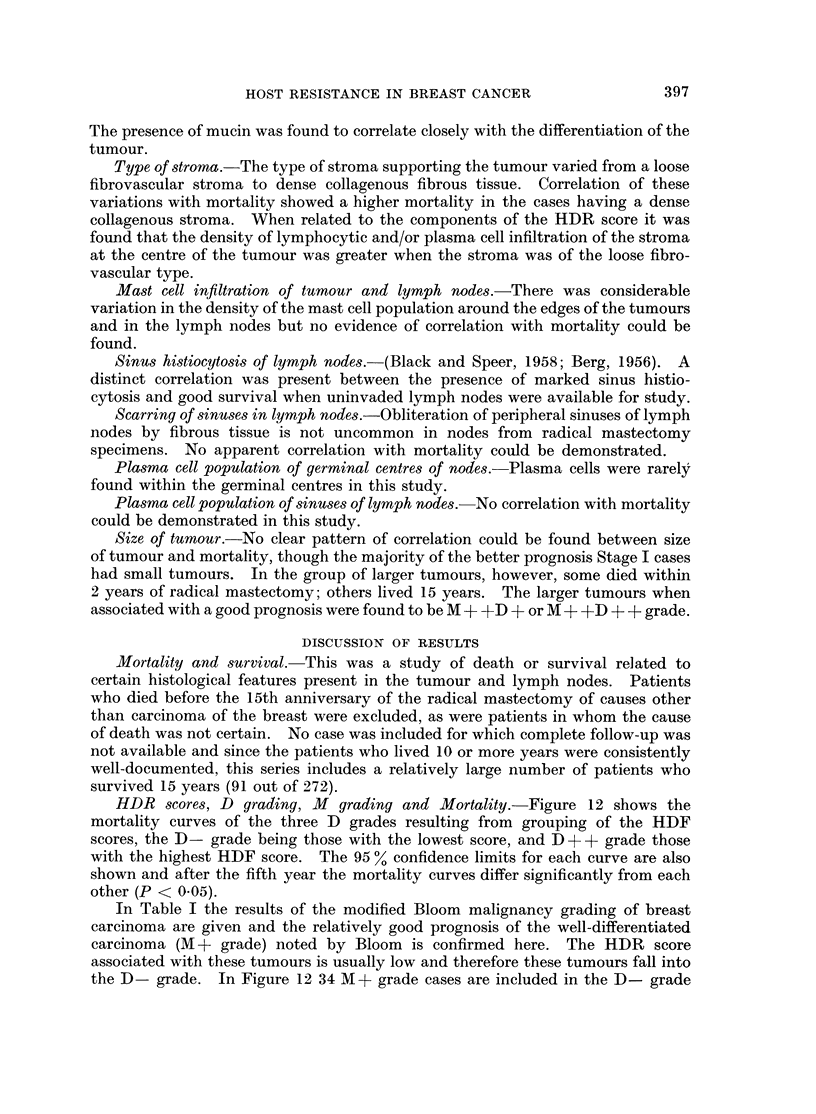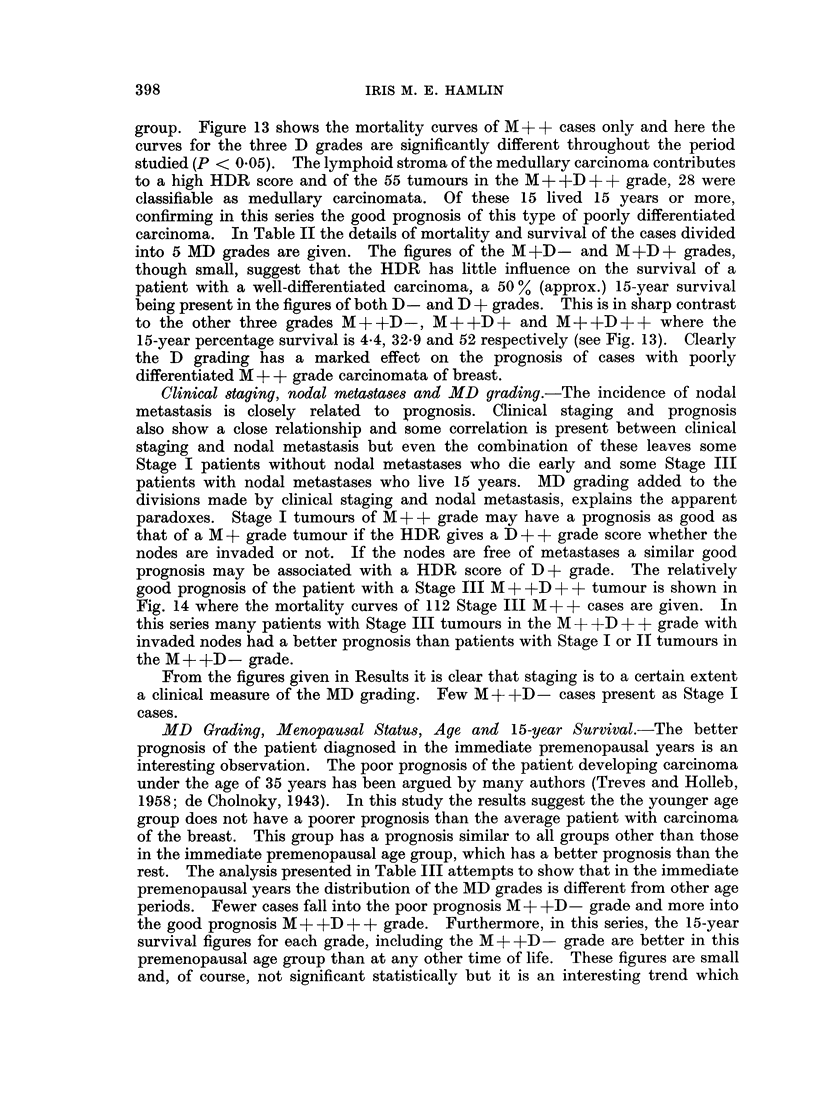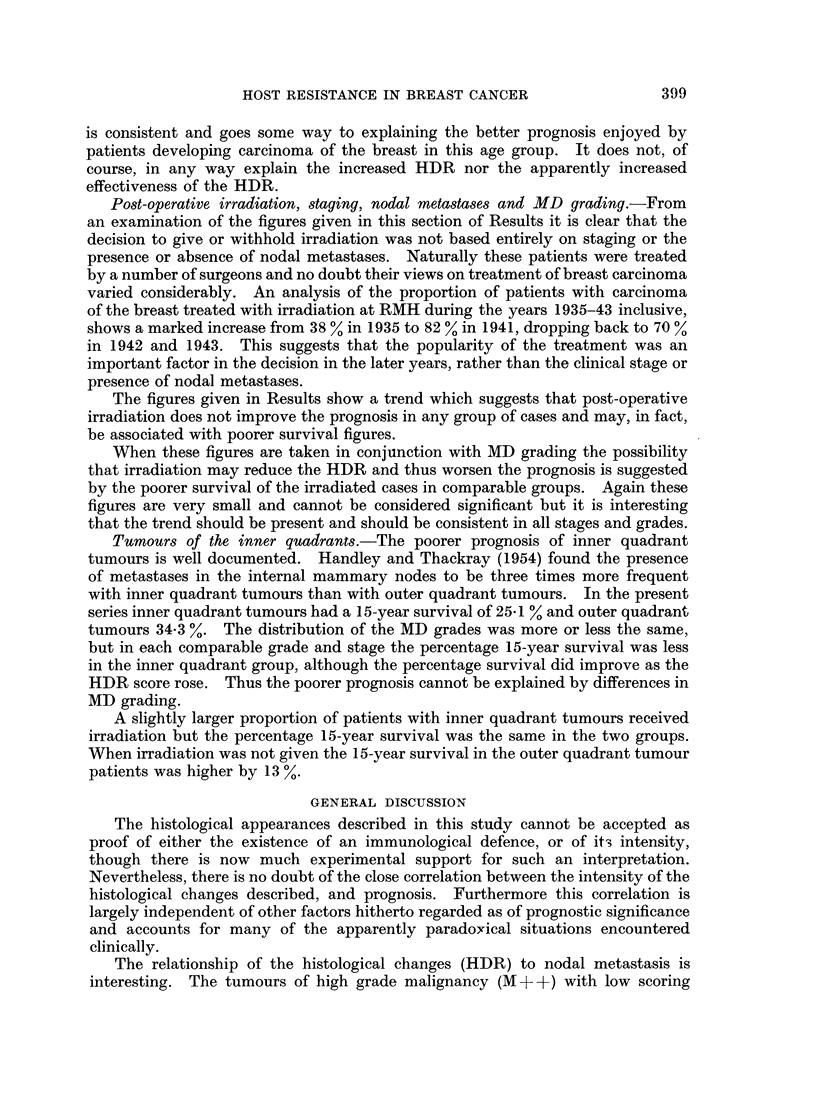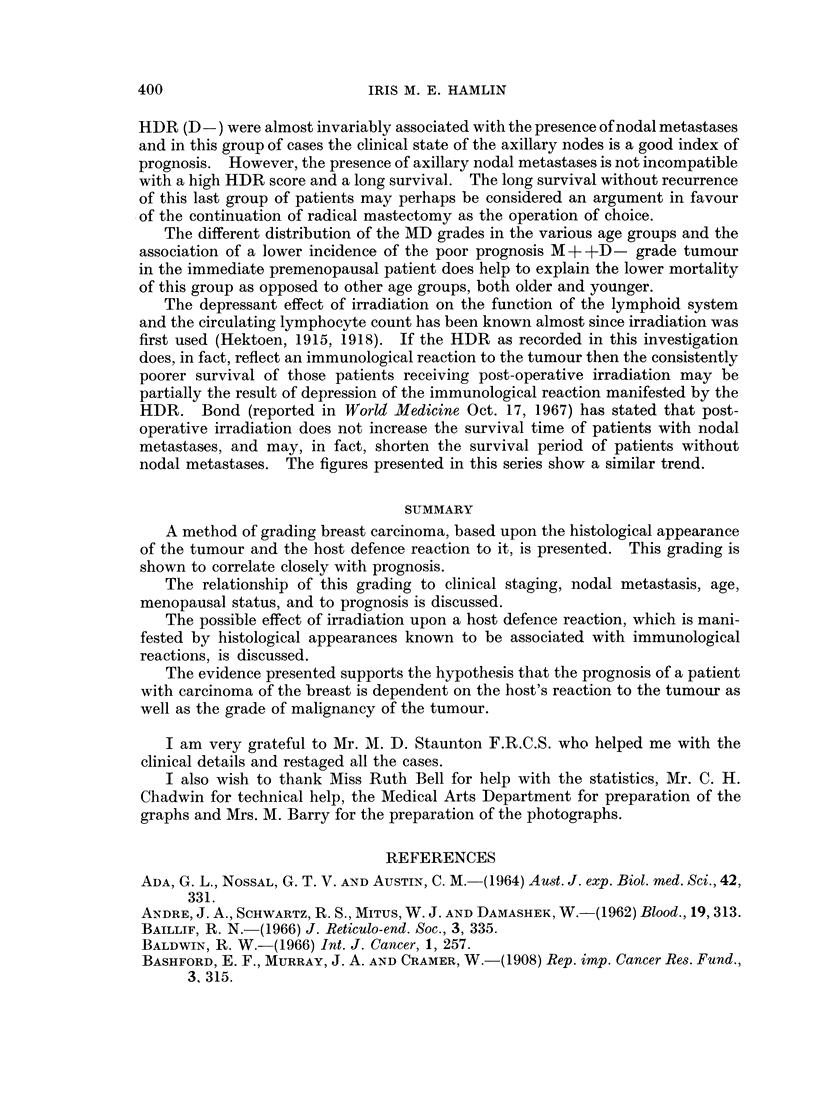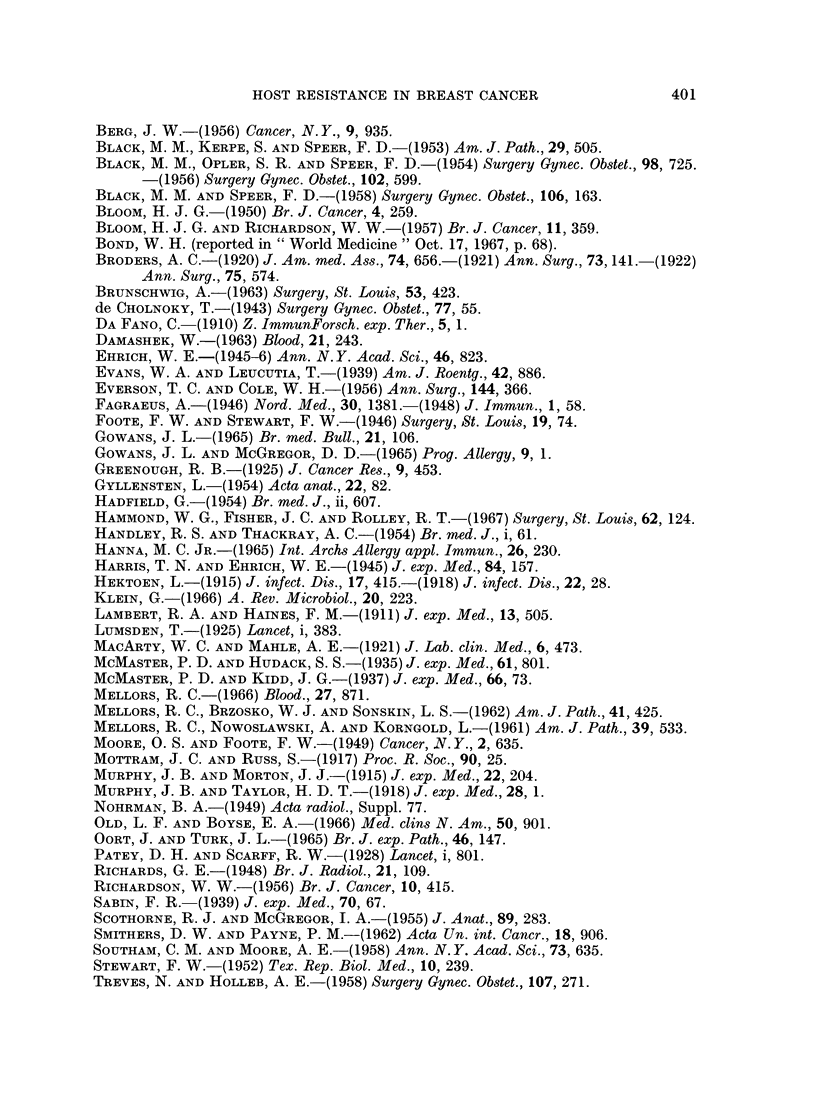# Possible host resistance in carcinoma of the breast: a histological study.

**DOI:** 10.1038/bjc.1968.47

**Published:** 1968-09

**Authors:** I. M. Hamlin

## Abstract

**Images:**


### BRITISH JOURNAL OF CANCER

VOL. XXII SEPTEMBER, 1968 NO. 3

### POSSIBLE HOST RESISTANCE IN CARCINOMA OF THE BREAST: A HISTOLOGICAL STUDY

IRIS M. E. HAMLIN

*From the Royal Marsden Hospital, London, S.W.3*

Received for publication May 17, 1968

THIS investigation was undertaken to test the hypothesis that the body has a defence mechanism against tumour invasion and spread, and that this defence has failed in patients who die with widespread metastasis. The unpredictability of tumour prognosis and the occasional case of undoubted spontaneous regression of tumour (Stewart, 1952; Everson and Cole, 1956; Brunschwig, 1963) could be explained by such a mechanism.

Although the antigenicity of human tumours is still not absolutely proved there is much evidence to suggest that certain malignant cells can act as antigens (Southam and Moore, 1958), and experimental workers have definite evidence of antigenic activity in many induced and spontaneous tumours in animals (Old and Boyse, 1966; Klein, 1966; Baldwin, 1966; Hammond, Fisher and Rolley, 1967). It therefore seems reasonable to assume that if a defence mechanism does exist it will be, at least in part, immunological. It is impossible in retrospect (and at present in prospect) to measure the antigenic power of a tumour but certain histological appearances are known to be associated with immunological responses in experimental animals (Scothorne and McGregor, 1955; Andre *et al.*, 1962; Oort and Turk, 1965) and have been observed in man in similar (but non-experimental) conditions (Mellors, Brzosko and Sonskin, 1962; Mellors, Nowoslawski and Korngold, 1961). In this study these features are accepted as possible evidence of immunological reaction, and an attempt is made to assess the intensity of the reaction from the degree of histological change. If these features reflect the presence of an immunological reaction which acts as a defence mechanism, and the histological appearances can be graded according to intensity, then the results should have prognostic significance, provided that other factors which may influence the immunological reactivity of the body remain the same.

Much work has been done on the prognostic significance of the grade of malignancy of tumours based on the degree of differentiation, beginning with Broders' studies of squamous cell carcinoma in 1920 (Broders, 1920, 1921, 1922). This type of grading was applied to breast carcinoma by Greenough in 1925, Scarff in 1928 (Patey and Scarff, 1928) and later by Bloom in 1950. Bloom's grading of breast carcinoma explains the rapid decline of those patients with small but highly malignant undifferentiated tumours without axillary metastases, who die in the first few years after radical mastectomy, but it does not explain the apparently paradoxical long survival of the patient who has a highly malignant undifferentiated tumour with metastatic deposits in axillary nodes. Something other than the grade of malignancy of the tumour must be invoked to explain these contradictory findings. Again, metastases may manifest themselves at any time up to 20 years or more after radical mastectomy. Clearly some factor inhibits the growth of viable tumour cells for a variable length of time (Hadfield, 1954). One possible factor is host resistance.

Host resistance has been discussed in cancer literature for half a century (Bashford, Murray and Cramer, 1908; Da Fano, 1910; Lambert and Haines, 1911; Murphy and Morton, 1915; Mottram and Russ, 1917; Murphy and Taylor, 1918; McArty and Mahle, 1921; Lumsden, 1925), and in 1946 Foote and Stewart suggested that the lymphocytic infiltration of the stroma of medullary carcinoma might represent a host reaction to the presence of tumour. A good prognosis is now known to be associated with this type of breast carcinoma (Moore and Foote, 1949; Richardson, 1956), and long survival has been shown to be associated with lymphocytic infiltration of the primary tumour and reactive changes in the draining lymph nodes of breast and stomach carcinomata (Black, Kerpe and Speer, 1953; Black, Opler and Speer, 1954, 1956). Recently the nature of the resistance has been specified as probably immunological. It has been known for a long time that the lymphoid system is the major tissue of reactivity to antigen (McMaster and Hudack, 1935; McMaster and Kidd, 1937; Sabin, 1939; Harris and Ehrich, 1945; Ehrich, 1945; Gyllensten, 1954) and since 1946 the plasma cell has been known to be involved in the production of circulating antibodies (Fagraeus, 1946, 1948). More recently much of the mystery of the function of the lymphocyte has been solved (Gowans and McGregor, 1965) and the immunological importance of it and of lymph node activity has been demonstrated (Ada, Nossal and Austin, 1964; Gowans, 1965; Hanna, 1965; Baillif, 1966). Although histological appearances can give no clue to the mechanism of a host defence reaction, the presence of histological features associated with known immunological reactions if correlated with survival may be taken as presumptive evidence of an immunological reaction which is functioning as a host defence.

Radical mastectomy specimens with the tumour and the regional lymph nodes removed at one operation provide ideal pathological material for histological assessment of tumour and lymph node appearances and an analysis was planned in which the grade of tumour malignancy and the intensity of the reaction to the tumour, both in the breast and in the axillary lymph nodes, was recorded. In order to present in numerical form an overall picture of the host defence reaction, scores indicating the density of lymphocytes and plasma cells in and around the tumour, and the intensity of the reaction in the nodes, were added together, the scoring being done in such a way that a high score denoted an intense reaction and vice versa. This score was then correlated with the grade of malignancy of the tumour and the survival time. Other factors, such as clinical stage, site of tumour, age of patient, etc. (de Cholnoky, 1943; Richards, 1948; Nohrman, 1949; Handley and Thackray, 1954; Bloom and Richardson, 1957; Treves and Holleb, 1958; Smithers and Payne, 1962) considered to influence mortality and survival were also recorded and included in the final correlation, to discover whether the presumptive immunological reaction had prognostic significance in its own right.

### MATERIAL

In such an investigation as this it is essential to have no knowledge of the fate of the patient when making the histological assessment of the tumour and the nodes. The primary selection was therefore made on the basis of pathological material available. All Royal Marsden Hospital (RMH) cases of radical mastectomy carried out between 1935 and 1942 for which blocks were extant were studied. Sections were cut and stained for histological assessment of the tumour and lymph nodes (see under Methods). Unfortunately the clinical notes of at least one third of the cases showed that the patients had been " lost to follow-up ". To replace these lost cases, RMH radical mastectomy cases in 1943, 1944 and 1945, for which it was known that there were complete clinical notes and paraffin blocks, were added. Apart from this there was no conscious selection of the cases analysed.

Of the total of 360 fully documented cases 20 cases had to be discarded because irradiation had been given *before* radical mastectomy. Since irradiation produces marked changes in lymph nodes any assessment made after irradiation could have no value as an index of host reaction. A further 68 cases were excluded because death had occurred within the 15-year follow-up period from diseases unrelated to the breast carcinoma. Since this was a study of death or survival related to certain histological features it was essential to know that when death occurred, death was due to carcinoma. Finally 272 cases, either dying of carcinoma before 15 years or living for 15 years or more, remained for analysis.

### METHODS

### *Grading of Intensity of " Host Defence Reaction "*

For each case blocks of the tumour and of the axillary nodes were recut and new sections stained with methyl green-pyronin to demonstrate the presence of plasma cells and other pyroninophilic cells.

#### *Cellular infiltration around the tumour*

The sections of the tumour were examined and the cellular infiltration around the tumour was assessed under the following headings: (1) pattern and density of infiltration, (2) density of plasma cell infiltration, (3) density of lymphocytic infiltration, (4) presence of, and density of, large immature pyroninophilic reticulum cells, sometimes called " immunoblasts " (Damashek, 1963; Mellors, 1966). Each factor was scored 0–3. Of these 4, only factor (1) requires further explanation. Lymphocytic and plasma cell infiltration is frequently seen around the periphery of the tumour but varies greatly in density and pattern. In some tumours a wide zone of cellular infiltration surrounds the whole of the edge of the tumour; in others only a few small collections of lymphocytes are present at widely spaced intervals around the periphery, and, of course, all intermediate stages are seen. The density of infiltration usually correlates with pattern but a very thinly populated zone of cells completely surrounding the tumour is possible. The patterns and densities were grouped and scored 0–3 (see below). Because factors (2), (3), and (4) were qualifying factors of factor (1) all the scores were added together and the average of the sum gave a possible final score of from 0 to 3.

*Cellular infiltration of centre of the tumour*

The cellular infiltration in the substance of the tumour was analysed on 2 factors only: (1) Density of lymphocytes, scored 0–3: (2) Density of plasma cells and/or large pyroninophilic cells scored 0–3: in practice only plasma cells were found in this position. The sums of (1) and (2) were averaged.

*Lymph nodes*

The appearances in the lymph nodes were analysed under the following headings: (1) follicular hyperplasia, (2) reticulum cell hyperplasia, (3) plasma cells in the medulla, (4) pyroninophilia of the large central cells of germinal centres, (5) large pyroninophilic reticulum cells in cortex. The scores registered under follicular hyperplasia and pyroninophilia of germinal centres ran parallel and so (in 99% of cases) did scores for (2) and (5). In both cases these scores were averaged. Uninvaded nodes were used for this analysis where possible and except for 6 cases enough nodal tissue remained even in invaded nodes, to record the factors listed above.

### *Details of Coding Related to Scoring*

*Cellular infiltration around the tumour*

| (1) *Pattern and density of infiltration* | *Score* |
|---|---|
| No focal areas of lymphocytic infiltration present, and only an occasional lymphocyte or plasma cell seen (Fig. 1). | 0 |
| Small scattered focal areas of lymphocytic infiltration present or a very thin entire zone of lymphocytes present (Fig. 2). | 1 |
| Numerous larger focal areas of lymphocytic infiltration present with moderate number of plasma cells, an occasional " immunoblast " or one germinal centre (Fig. 3). | 2 |
| Entire zone of lymphocytes, plasma cells and pyroninophilic reticulum cells surrounding the tumour or very numerous large focal areas of lymphocytic infiltration with plasma cells and/or germinal centres (Fig. 4, 5). | 3 |

---

EXPLANATION OF PLATES

FIG. 1.—Edge of tumour showing sparse lymphocytic infiltration. ×25.
FIG. 2.—Edge of tumour showing small scattered focal areas of lymphocytic infiltration. ×25.
FIG. 3.—Edge of tumour showing numerous larger focal areas of lymphocytic infiltration. ×25.
FIG. 4.—Edge of tumour showing entire wide zone of lymphocytes and plasma cells. ×75.
FIG. 5.—High power of cellular infiltration present in Fig. 4 to show presence of an immunoblast. ×300.
FIG. 6.—Centre of tumour with dense lymphocytic and plasmacytic infiltration of the stroma between the groups of carcinoma cells. ×60.
FIG. 7.—High power of Fig. 6. ×250.
FIG. 8.—Portion of a lymph node in which no germinal centres were found. ×60.
FIG. 9.—Portion of a lymph node in which reactive changes were marked. ×60.
FIG. 10.—Pyroninophilic cell " immunoblast " in the cortex of a lymph node. ×250.
FIG. 11.—Well differentiated M+ (Bloom Grade I) carcinoma of breast. ×60.

1 2 3

Hamlin.

4

5

Hamlin.

6

7

8

Hamlin.

9

10

11

| | Score |
|---|---|
| (2) *Plasma cells* | |
| (Numbers per High Power Field) | |
| 0 | 0 |
| 0·5–2 per HPF | 1 |
| 3–10 per HPF | 2 |
| More than 10 per HPF | 3 |
| (3) *Lymphocytic infiltration* | |
| Absent | 0 |
| Present + | 1 |
| Present ++ | 2 |
| Present +++ | 3 |
| (4) *Pyroninophilic cells* (other than plasma cells) | |
| None seen | 0 |
| Up to 3 seen (especially in focal lymphocytic areas) | 1 |
| 3–10 seen | 2 |
| More than 10, or a germinal centre showing marked pyroninophilia | 3 |

*Cellular infiltration of centre of the tumour* (Fig. 6, 7)

| | |
|---|---|
| (1) *Density of lymphocytes in the tumour* | |
| Absent | 0 |
| Present + | 1 |
| Present + | 2 |
| Present +++ | 3 |
| (2) *Density of plasma cells and pyroninophilic cells in the tumour* | |
| Absent | 0 |
| 0·5–10 per HPF | 1 |
| 10–20 per HPF | 2 |
| More than 20 per HPF | 3 |

*Lymph Nodes*

| | |
|---|---|
| (1) *Follicular hyperplasia* | |
| Germinal centres absent (Fig. 8) | 0 |
| Germinal centres just recognisable | 0·5 |
| Germinal centres present + | 1 |
| Germinal centres present ++ | 1·5 |
| Germinal centres present +++ (Fig. 9). | 2 |
| (2) *Reticulum cell hyperplasia* | |
| No reticulum cells seen | 0 |
| Very few reticulum cells present | 0·5 |
| Moderate number reticulum cells present | 1 |
| Numerous reticulum cells | 2 |

| (3) *Plasma cells in medulla and cortex* | *Score* |
|---|---|
| No plasma cells seen | 0 |
| Scanty plasma cells present | 0·5 |
| Moderate numbers in medulla, in perisinusoidal position | 1 |
| Numerous groups, or diffuse infiltration throughout node | 2 |
| (4) *Pyroninophilia of germinal centres* | |
| Germinal centres absent | 0 |
| Few pyroninophilic cells | 0·5 |
| Germinal centres present, pyroninophilic cells + | 1 |
| Germinal centres present, pyroninophilic cells ++ | 1·5 |
| Germinal centres present, pyroninophilic cells +++ | 2 |
| (5) *Pyroninophilic cells (other than plasma cells) in pulp* (Fig. 10). | |
| No pyroninophilic cells seen | 0 |
| An occasional pyroninophilic reticulum cell present | 0·5 |
| Moderate number of pyroninophilic reticulum cells present | 1 |
| Numerous pyroninophilic reticulum cells present | 2 |

The maximum possible final score for the periphery of the tumour is 3, for the centre of the tumour is 3, and for the lymph nodes is 6. The maximum possible *total* score is 12 and indicates the most intense reaction. This score of host defence reaction (HDR) is used in shortened form in the tables and script as D−, D+, and D++ (see under Results).

### *Grading of Malignancy of the Tumour*

The grade of malignancy of the tumour was assessed on the histological criteria laid down by Greenough (1925), Patey and Scarff (1928) and Bloom (1950), but when correlated with mortality and drawn in graph form there was no clear division in this study on grouping of scores above 4. A two-grade malignancy (M+ and M++) was therefore adopted in this series—those cases scoring 3 or 4 being put into the M+ grade (Fig. 11), i.e. almost equivalent to Grade I of Bloom, and all scores higher than 4 into M++ grade; thus M+ grade includes all well-differentiated tumours and M++ grade the moderately differentiated and undifferentiated tumours.

### *Other Histological Features Recorded*

These included the type of tumour, i.e. whether infiltrating carcinoma, intraduct carcinoma, intralobular carcinoma or mucoid carcinoma, the presence of mucin in tumour, the type of stroma supporting the tumour, e.g. fibrovascular or dense collagen, the mast cell infiltration of the tumour and lymph nodes, sinus histiocytosis of lymph nodes, scarring of sinusoids in lymph nodes, plasma cell population of germinal centres of nodes, plasma cell population of sinusoids of nodes, and presence or absence of metastatic tumour in nodes.

### *Other Data*

When the histological analysis was complete the following data were recorded: age, menopausal status, site and size of the tumour in breast, and clinical stage of the tumour (clinical staging was done afresh from the clinical notes using standards of the current TNM method), details of irradiation therapy, and whether alive or dead with date and cause of death.

### RESULTS

All except one of the tumours were infiltrating carcinomata. The exception was an intraduct carcinoma in the section available for study but since this tumour measured between 3 and 5 cm. and the clinical stage was given as II it was considered to be almost certainly infiltrating in some other area and the case was not excluded. This patient was alive without recurrence at 16 years.

*Mortality*.—Of the 272 cases analysed and followed up, 91 were alive without evidence of recurrence at 15 years; 31 were dead at the end of the 1st year, 79 by the end of the 2nd year. At the 5th anniversary of operation a total of 137 were dead and by the 10th anniversary a further 35 had died, leaving 9 cases who died between 10 and 15 years after operation.

*HDF scores and D grading*.—Analysis of the cases by HDF (see under Methods) and mortality, gives the results presented in Fig. 12. The total scores are divided into three groups:—

| | | | |
|---|---|---|---|
| Group 1 | HDF scores 0–4 | Grade " D− " | 102 cases |
| Group 2 | HDF scores 4·25–5·75 | Grade " D+ " | 110 cases |
| Group 3 | HDF scores 6 and over | Grade " D++ " | 60 cases |

*Malignancy grading (M+ and M++)*.—Table I shows the cases divided by malignancy grading (see under Methods).

TABLE I.—*Survival According to Malignancy Grading*

| Dead at | 1 yr. | 2 yrs. | 3 yrs. | 5 yrs. | 7 yrs. | 10 yrs. | 15 yrs. | Alive at 15 yrs. + | Total |
|---|---|---|---|---|---|---|---|---|---|
| M+ | 2 | 6 | 4 | 7 | 4 | 4 | 4 | 30 | 61 |
| M++ | 29 | 42 | 20 | 27 | 15 | 12 | 5 | 61 | 211 |

*M and D gradings combined*.—Analysis of 272 cases by both M and D grading shows 61 M+ cases to be divided between the three D groups as follows: D− 34, D+ 22, D++ 5. Since the D++ group was so small and the mortality figures similar to those of the D+ it was decided to have two M+ groups:—

M+D− = M scores 3 and 4 with D scores 0–4
M+D+ = M scores 3 and 4 with D scores above 4

In the 211 M++ cases i.e. those with M scores 5 and above, the three D groups were retained. Thus the five MD grades are M++D−, M++D+, M++D++, M+D−, M+D+. Table II gives the cases analysed by these grades and correlated with mortality. In Fig. 13 division of the 211 M++ cases by D grading correlated with mortality is shown in graph form.

*Nodal metastases*.—In many studies of breast carcinoma the prognosis has been shown to be greatly influenced by the presence or absence of axillary nodal metastases and staging is an attempt to assess this situation clinically. Analysis of the 272 cases shows axillary metastases present in 194 cases, and absent in 78

FIG. 12.—Graph to show mortality after radical mastectomy of 272 cases (M+ + and M+ grades) in three D grades.

($P$ < 0·05).

D 0–4 = D− grade
D 4·25–5·75 = D+ grade
D 6+ = D+ + grade

TABLE II.—*MD Grades and Mortality*

| Dead at | 1 yr. | 2 yrs. | 3 yrs. | 5 yrs. | 7 yrs. | 10 yrs. | 15 yrs. | Alive at 15 yrs. + | Total |
|---|---|---|---|---|---|---|---|---|---|
| M+ +D− | 19 | 15 | 8 | 10 | 8 | 4 | 1 | 3 | 68 |
| M+ +D+ | 9 | 18 | 8 | 11 | 6 | 5 | 2 | 29 | 88 |
| M+ +D+ + | 1 | 9 | 4 | 6 | 1 | 3 | 2 | 29 | 55 |
| M+D− | 2 | 3 | 3 | 2 | 3 | 2 | 1 | 18 | 34 |
| M+D+ | 0 | 3 | 1 | 5 | 1 | 2 | 3 | 12 | 27 |

cases. Of the 194 cases with axillary metastases, 41 were alive 15 years or more, 121 were dead at the end of 5 years. Of the 78 cases without axillary metastases 50 were alive 15 years or more and only 16 were dead at the end of 5 years.

*Clinical staging and nodal metastases.*—When clinical staging is used as the basis of analysis, 59 cases fall into Stage I, 76 into Stage II, 137 into Stage III.

FIG. 13.—Graph to show mortality after radical mastectomy of 211 cases of M++ grade in three D grades

($P < 0{\cdot}05$)

D 0–4 = D− grade
D 4·25–5·75 = D+ grade
D 6+ = D++ grade

36 Stage I cases, 32 Stage II cases and 23 Stage III cases were alive 15 years or more after operation.

Histological evidence of metastasis was found in the lymph nodes from the radical mastectomy specimens of 27 patients who had been staged clinically Stage I; of these 27, 10 lived 15 years or more. In Stage II cases nodal metastases were found in 53 cases of whom 17 lived 15 years or more, and were absent in 23 cases, 15 of whom lived 15 years or more. Of 114 Stage III cases showing evidence of nodal metastasis, only 14 cases lived 15 years or more; 23 cases were without evidence of metastasis and 9 of these lived 15 years or more.

*Clinical staging and MD grading.*—Analysis of 211 M++ cases shows that of the 39 M++ cases in Stage I, 9 are D− grade, 22 are D+ grade and 8 are D++ grade. One of the 9 cases graded D− (11·1 %), 13 of the 22 D+ cases (59·09 %) and 7 of the 8 (87·5 %) D++ cases were alive at 15 years.

In Stages II and III similar trends in the figures are found. In Stage II 15 cases are D− grade and 1 was alive at 15 years (6·6 %); 24 cases are D+ grade and 10 were alive at 15 years (41·6 %); 21 cases are D++ and 11 were alive at 15 years (52·3 %). In Stage III 44 cases are D− grade, of whom only 1 was alive at 15 years (2·2 %); 42 cases are D+ with 6 alive at 15 years (14·2 %); 26 cases are D++ with 11 alive at 15 years (42·3 %). Figure 14 gives in graph form the mortality of 112 M++ Stage III cases divided by D grading. ($P <$ or approx. = to 0·05).

FIG. 14.—Graph to show mortality after radical mastectomy of 112 Stage III cases of M++ grade in three D grades

($P <$ or approx. = to 0·05)

D 0–4 = D− grade
D 4·25–5·75 = D+ grade
D 6+ = D++ grade

Analysis of 61 M+ cases by staging and D grading gives the following 15-year survival figures:—

| | | |
|---|---|---|
| Stage I | D− | 8 out of 10 cases alive 15 years. |
| | D+ and D++ | 7 out of 10 cases alive 15 years. |
| Stage II | D− | 7 out of 11 cases alive 15 years. |
| | D+ and D++ | 3 out of 5 cases alive 15 years. |
| Stage III | D− | 3 out of 13 cases alive 15 years. |
| | D+ and D++ | 2 out of 12 cases alive 15 years. |

*Clinical staging, MD grading and nodal metastases.*—Additional analysis of nodal metastases in Stages I, II and III gives the following 15-year survival figures:—

| | | Number alive 15 yrs, or more | |
|---|---|---|---|
| | | +ve Nodes | −ve Nodes |
| Stage I | | | |
| | M++D− | . 0 out of 3 | 0 out of 2 |
| | M++D+ | . 1 out of 8 | 11 out of14 |
| | M++D++ | . 3 out of 6 | 6 out of 6 |
| | M+D− | . 3 out of 4 | 3 out of 4 |
| | M+D+ | . 3 out of 6 | 6 out of 6 |
| Stage II | | | |
| | M++D− | . 1 out of 11 | No cases |
| | M++D+ | . 5 out of 18 | 3 out of 6 |
| | M++D++ | . 6 out of 14 | 7 out of 10 |
| | M+D− | . 2 out of 5 | 4 out of 4 |
| | M+D+ | . 3 out of 6 | 2 out of 2 |
| Stage III | | | |
| | M++D− | . 1 out of 29 | 0 out of 2 |
| | M++D+ | . 4 out of 41 | 2 out of 7 |
| | M++D++ | . 7 out of 25 | 4 out of 8 |
| | M+D− | . 1 out of 7 | 1 out of 2 |
| | M+D+ | . 1 out of 12 | 2 out of 4 |

*Menopausal status and* 15-*year survival.*—The better prognosis of the patient in the immediate premenopausal years has been shown by a number of authors (Evans and Leucutia, 1939; Richards, 1948; Nohrman, 1949; Smithers and Payne, 1962). Analysis of the 272 cases by menopausal status and mortality confirmed these findings. Ninety-six patients were premenopausal; 41 lived 15 years or more without recurrence (42·7 %). Fifteen patients were recorded as being menopausal; 6 lived 15 years or more (40 %). Of 131 post-menopausal patients only 32 lived 15 years or more (24·4 %).

Division of the cases into definite age groups gave the following 15-year survival figures: 33·3 % in the 30–39 age group, 37·1 % in the 40–44 age group, 47 % in the 45–49 age group, 31·2 % in the 50–59 age group, 20·5 % in the 60–69 age group and 29·4 % in the 70 and over age group. The highest 15-year survival figure occurs in the 45–49 age group.

*MD grade, menopausal status, age and* 15-*year survival.*—Analysis of the figures in each age group by MD grading shows that the proportion of cases in each MD grade varies with the age group. In 45–49 age group only a small proportion of cases fall into the M++D− grade which has such a poor prognosis. A high proportion are in M++D+, and the rest divided evenly between the other three grades M++D++, M+D−, M+D+. In the 50–59 age group by far the largest number are of M++D− grade and of these only one was alive 15 years. In other age groups the division of the cases between the grades is intermediate between these extremes.

The fifteen-year survival of the cases in the various grades in the different age groups also varies; of 20 cases in M++D+ grade in the 45–49 age group 10 cases lived 15 years or more (50 %), of 9 M++D+ cases in the 30–39 age group 2 lived 15 years (22·2 %). A similar lower 15-year survival figure is present in the 50–59 years group and 60–69 years group.

Table III attempts to present these variations in the six age group divisions.

TABLE III.—*Fifteen Year Survival by Grades and Age Groups*

| Age Group | * | MD Grades M++D− | M++D+ | M++D++ | M+D− | M+D+ | % 15-yr. survival in age group |
|---|---|---|---|---|---|---|---|
| 30 to 39 yrs. | A | 4 | 9 | 3 | 4 | 1 | 33·3 |
| | B | 19·0 | 42·3 | 14·2 | 19·0 | 4·5 | |
| | C | 0 | 2 | 2 | 3 | 0 | |
| | D | 0 | 22·2 | 66·6 | 75 | 0 | |
| | E | 0 | 9·5 | 9·5 | 14·2 | 0 | |
| 40 to 44 yrs. | A | 6 | 12 | 6 | 5 | 6 | 37·1 |
| | B | 17 | 34 | 17·1 | 14·2 | 17·1 | |
| | C | 0 | 5 | 2 | 2 | 4 | |
| | D | 0 | 41·2 | 33·3 | 40 | 66·6 | |
| | E | 0 | 14·2 | 5·6 | 5·6 | 11·4 | |
| 45 to 49 yrs. | A | 5 | 20 | 11 | 9 | 6 | 47·0 |
| | B | 9 | 39·2 | 21·5 | 17·6 | 11·7 | |
| | C | 1 | 10 | 5 | 6 | 3 | |
| | D | 20 | 50 | 45·5 | 66·6 | 50 | |
| | E | 1·9 | 19·6 | 9·8 | 11·7 | 5·8 | |
| 50 to 59 yrs. | A | 29 | 21 | 19 | 6 | 5 | 31·2 |
| | B | 36·2 | 26·2 | 23·7 | 7·5 | 6·2 | |
| | C | 1 | 5 | 13 | 4 | 2 | |
| | D | 3·4 | 24·0 | 68·3 | 66·6 | 40 | |
| | E | 1·3 | 6·2 | 16·2 | 5 | 2·5 | |
| 60 to 69 yrs. | A | 17 | 22 | 11 | 10 | 8 | |
| | B | 25·5 | 32·3 | 16·1 | 14·5 | 11·7 | 20·5 |
| | C | 1 | 5 | 4 | 3 | 3 | |
| | D | 5·8 | 23 | 36·3 | 30 | 37·5 | |
| | E | 1·4 | 7 | 5·8 | 4·4 | 4·4 | |
| 70+ yrs. | A | 8 | 3 | 5 | 1 | 0 | 29·4 |
| | B | 47·0 | 17·1 | 29·3 | 5·8 | 0 | |
| | C | 0 | 2 | 3 | 0 | 0 | |
| | D | 0 | 66·6 | 60·0 | 0 | 0 | |
| | E | 0 | 11·7 | 17·8 | 0 | 0 | |

* A = Number of cases in each MD grade in each age group.
B = Number of cases (A) expressed as % of total number of cases in age group.
C = Number of cases in each grade and age group alive 15 yrs. or more.
D = Number of cases alive 15 yrs. or more (C) expressed as a % of (A).
E = Number of cases alive (C) expressed as % of total cases in age group.

*Post-operative irradiation and 15-year survival.*—One hundred and eighty four patients received post-operative irradiation, 88 did not. Of the 184 patients, 44 lived 15 years (23·8 %) or more; 47 of the 88 patients lived 15 years or more (53·3 %).

*Post-operative irradiation, staging, nodal metastases and 15-year survival.*—Division of the cases by staging and post-operative irradiation shows an almost equal division of 59 Stage I cases, 30 receiving irradiation. In Stage II, 51 cases out of 76 received irradiation and in Stage III, 103 out of 137.

Further analysis of these figures by nodal metastases shows that of the 27 with metastases in Stage I 16 received post-operative irradiation, 11 did not. Of the 16 irradiated cases, 3 lived 15 years or more (18·7 %), of the 11 non-irradiated cases, 7 lived 15 years or more (63·6 %). Fourteen cases in Stage I in which nodal metastases were not found received irradiation, of whom 10 were alive 15 years or more (68·5 %). Eighteen Stage I cases with negative nodes did not receive irradiation; 16 of these were alive 15 years or more (88·8 %).

In Stages II and III a similar trend is evident (Tables IV, V).

TABLE IV.—*Post-operative Irradiation, Nodal Metastases and Survival in Stage II*

| | Nodes +ve | DXR given | DXR not given | | Nodes −ve | DXR given | DXR not given |
|---|---|---|---|---|---|---|---|
| | 54 | 39 | 15 | | 22 | 12 | 10 |
| Alive 15 yrs. + | 17 | 12 | 5 | Alive 15 yrs. + | 16 | 7 | 9 |
| % alive 15 yrs. + | 31·4 | 30·8 | 33·3 | % alive 15 yrs. + | 72·2 | 59·3 | 90 |

TABLE V.—*Post-operative Irradiation, Nodal Metastases and Survival in Stage III*

| | Nodes +ve | DXR given | DXR not given | | Nodes −ve | DXR given | DXR not given |
|---|---|---|---|---|---|---|---|
| | 114 | 90 | 24 | | 23 | 13 | 10 |
| Alive 15 yrs. + | 14 | 9 | 5 | Alive 15 yrs. + | 9 | 4 | 5 |
| % alive 15 yrs. + | 12·2 | 10 | 20·8 | % alive 15 yrs. + | 39·1 | 37·6 | 50 |

*Post-operative irradiation, staging, nodal metastases, MD grading and 15-year survival.*—When the figures given above under staging, nodal metastases, and MD grading, are further divided into those receiving and not receiving post-operative irradiation, the following figures are obtained:—

Stage I M++D− The 5 patients in M++D− grade were dead before 15 years. Four received irradiation, 2 of these having invaded nodes.

M++D+ Seven patients with nodal metastases receiving irradiation died before 10 years.

One patient not receiving DXR lived over 15 years. Of 14 patients with negative nodes, 5 received irradiation and 9 did not. Four of those receiving irradiation and 7 of those who did not receive irradiation lived more than 15 years.

M++D++ Six patients with nodal metastases. Five received irradiation and 2 of these lived 15 years; 1 did not and was alive after 15 years. In 6 patients the nodes were free of metasteses, 2 received irradiation: all 6 lived.

M+D− Eight patients, 4 with nodal metastases, 4 without. Two of each group received irradiation. All 4 which did not receive irradiation lived 15 years or more. One with invaded nodes and one with uninvaded nodes given irradiation lived 15 years or more.

M+D+ Six patients with nodal metastases were not given irradiation, 3 lived 15 years or more. Six patients without nodal metastases, 3 received irradiation, all 6 were alive at 15 years.

TABLE VI.—*Post-operative Irradiation, Nodal Metastases, MD grading and Survival in Stage II*

| MD Grade | Nodes +ve | DXR given | Alive 15 yrs. + | DXR not given | Alive 15 yrs. + | Nodes −ve | DXR given | Alive 15 yrs. + | DXR not given | Alive 15 yrs. + |
|---|---|---|---|---|---|---|---|---|---|---|
| M++D− | 11 | 10 | 1 | 1 | 0 | . | . | . | . | . |
| M++D+ | 18 | 12 | 4 | 6 | 1 | 6 | 5 | 2 | 1 | 1 |
| M++D++ | 14 | 11 | 4 | 3 | 2 | 10 | 3 | 1 | 7 | 6 |
| M+D− | 6 | 2 | 1 | 3 | 1 | 4 | 3 | 3 | 1 | 1 |
| M+D+ | 6 | 4 | 2 | 2 | 1 | 2 | 1 | 1 | 1 | 1 |

TABLE VII.—*Post-operative Irradiation, Nodal Metastases, MD grading and Survival in Stage III*

| MD Grade | Nodes +ve | DXR given | Alive 15 yrs. + | DXR not given | Alive 15 yrs. + | Nodes −ve | DXR given | Alive 15 yrs. + | DXR not given | Alive 15 yrs. + |
|---|---|---|---|---|---|---|---|---|---|---|
| M++D− | 29 | 25 | 1 | 4 | 0 | 2 | 1 | 0 | 1 | 0 |
| M++D+ | 41 | 29 | 1 | 12 | 3 | 7 | 4 | 1 | 3 | 1 |
| M++D++ | 25 | 19 | 5 | 6 | 2 | 8 | 4 | 2 | 4 | 2 |
| M+D− | 7 | 7 | 1 | 0 | 0 | 2 | 2 | 1 | 0 | 0 |
| M+D+ | 12 | 10 | 1 | 2 | 0 | 4 | 2 | 0 | 2 | 2 |

*Tumours of inner quadrants.*—Comparison of inner and outer quadrant tumours shows a higher mortality with a larger number of deaths in the early years in Stages II and III tumours of the inner quadrants. In Stage I the 15-year survival figures are lower in inner quadrant tumours. The incidence of post-operative irradiation is higher for inner quadrant tumours, but the 15-year survival figures are similar in both inner and outer quadrant tumour cases when irradiation was given, viz. 20 %. The 15-year survival figure of non-irradiated outer quandrant tumours is considerably higher than that of the non-irradiated inner quadrant tumours. The distribution of the MD grades is similar in both groups and the incidence of positive nodes in the patients who lived 15 years is approximately the same.

TABLE VIII.—*Comparative Survival for Patients with Tumours of the Inner and Outer Quadrants*

| | Inner Quadrants | | | | | Outer Quadrants | | | | |
|---|---|---|---|---|---|---|---|---|---|---|
| | | | Alive 15 yrs. | | | | | Alive 15 yrs. | | |
| Stage | DXR+ | DXR− | DXR+ | DXR− | Total | DXR+ | DXR− | DXR+ | DXR− | Total |
| I | 6 | 5 | 2 | 4 | 11 | 18 | 22 | 8 | 17 | 40 |
| II | 16 | 5 | 6 | 2 | 21 | 33 | 15 | 6 | 9 | 48 |
| III | 25 | 9 | 2 | 1 | 34 | 60 | 18 | 9 | 8 | 78 |
| | 47 | 19 | 10 | 7 | 66 | 111 | 55 | 23 | 34 | 166 |
| Percentage surviving 15 years | | | | | 25·1 | | | | | 34·3 |
| Percentage of cases receiving irradiation | | | | | 71 | | | | | 60·8 |
| Percentage 15-year survival of patients receiving irradiation | | | | | 21 | | | | | 20·7 |
| Percentage 15-year survival of patients not receiving irradiation | | | | | 48·1 | | | | | 61·8 |

*Other data recorded* (see under Methods)

*Mucin production in the tumour.*—No tumours were entirely of the " colloid ". type though areas of " mucoid degeneration " were evident in some tumours.

The presence of mucin was found to correlate closely with the differentiation of the tumour.

*Type of stroma.*—The type of stroma supporting the tumour varied from a loose fibrovascular stroma to dense collagenous fibrous tissue. Correlation of these variations with mortality showed a higher mortality in the cases having a dense collagenous stroma. When related to the components of the HDR score it was found that the density of lymphocytic and/or plasma cell infiltration of the stroma at the centre of the tumour was greater when the stroma was of the loose fibrovascular type.

*Mast cell infiltration of tumour and lymph nodes.*—There was considerable variation in the density of the mast cell population around the edges of the tumours and in the lymph nodes but no evidence of correlation with mortality could be found.

*Sinus histiocytosis of lymph nodes.*—(Black and Speer, 1958; Berg, 1956). A distinct correlation was present between the presence of marked sinus histiocytosis and good survival when uninvaded lymph nodes were available for study.

*Scarring of sinuses in lymph nodes.*—Obliteration of peripheral sinuses of lymph nodes by fibrous tissue is not uncommon in nodes from radical mastectomy specimens. No apparent correlation with mortality could be demonstrated.

*Plasma cell population of germinal centres of nodes.*—Plasma cells were rarely found within the germinal centres in this study.

*Plasma cell population of sinuses of lymph nodes.*—No correlation with mortality could be demonstrated in this study.

*Size of tumour.*—No clear pattern of correlation could be found between size of tumour and mortality, though the majority of the better prognosis Stage I cases had small tumours. In the group of larger tumours, however, some died within 2 years of radical mastectomy; others lived 15 years. The larger tumours when associated with a good prognosis were found to be M+ +D+ or M+ +D+ + grade.

### DISCUSSION OF RESULTS

*Mortality and survival.*—This was a study of death or survival related to certain histological features present in the tumour and lymph nodes. Patients who died before the 15th anniversary of the radical mastectomy of causes other than carcinoma of the breast were excluded, as were patients in whom the cause of death was not certain. No case was included for which complete follow-up was not available and since the patients who lived 10 or more years were consistently well-documented, this series includes a relatively large number of patients who survived 15 years (91 out of 272).

*HDR scores, D grading, M grading and Mortality.*—Figure 12 shows the mortality curves of the three D grades resulting from grouping of the HDF scores, the D− grade being those with the lowest score, and D+ + grade those with the highest HDF score. The 95% confidence limits for each curve are also shown and after the fifth year the mortality curves differ significantly from each other ($P < 0{\cdot}05$).

In Table I the results of the modified Bloom malignancy grading of breast carcinoma are given and the relatively good prognosis of the well-differentiated carcinoma (M+ grade) noted by Bloom is confirmed here. The HDR score associated with these tumours is usually low and therefore these tumours fall into the D− grade. In Figure 12 34 M+ grade cases are included in the D− grade group. Figure 13 shows the mortality curves of M++ cases only and here the curves for the three D grades are significantly different throughout the period studied ($P < 0{\cdot}05$). The lymphoid stroma of the medullary carcinoma contributes to a high HDR score and of the 55 tumours in the M++D++ grade, 28 were classifiable as medullary carcinomata. Of these 15 lived 15 years or more, confirming in this series the good prognosis of this type of poorly differentiated carcinoma. In Table II the details of mortality and survival of the cases divided into 5 MD grades are given. The figures of the M+D− and M+D+ grades, though small, suggest that the HDR has little influence on the survival of a patient with a well-differentiated carcinoma, a 50% (approx.) 15-year survival being present in the figures of both D− and D+ grades. This is in sharp contrast to the other three grades M++D−, M++D+ and M++D++ where the 15-year percentage survival is 4·4, 32·9 and 52 respectively (see Fig. 13). Clearly the D grading has a marked effect on the prognosis of cases with poorly differentiated M++ grade carcinomata of breast.

*Clinical staging, nodal metastases and MD grading.*—The incidence of nodal metastasis is closely related to prognosis. Clinical staging and prognosis also show a close relationship and some correlation is present between clinical staging and nodal metastasis but even the combination of these leaves some Stage I patients without nodal metastases who die early and some Stage III patients with nodal metastases who live 15 years. MD grading added to the divisions made by clinical staging and nodal metastasis, explains the apparent paradoxes. Stage I tumours of M++ grade may have a prognosis as good as that of a M+ grade tumour if the HDR gives a D++ grade score whether the nodes are invaded or not. If the nodes are free of metastases a similar good prognosis may be associated with a HDR score of D+ grade. The relatively good prognosis of the patient with a Stage III M++D++ tumour is shown in Fig. 14 where the mortality curves of 112 Stage III M++ cases are given. In this series many patients with Stage III tumours in the M++D++ grade with invaded nodes had a better prognosis than patients with Stage I or II tumours in the M++D− grade.

From the figures given in Results it is clear that staging is to a certain extent a clinical measure of the MD grading. Few M++D− cases present as Stage I cases.

*MD Grading, Menopausal Status, Age and 15-year Survival.*—The better prognosis of the patient diagnosed in the immediate premenopausal years is an interesting observation. The poor prognosis of the patient developing carcinoma under the age of 35 years has been argued by many authors (Treves and Holleb, 1958; de Cholnoky, 1943). In this study the results suggest the the younger age group does not have a poorer prognosis than the average patient with carcinoma of the breast. This group has a prognosis similar to all groups other than those in the immediate premenopausal age group, which has a better prognosis than the rest. The analysis presented in Table III attempts to show that in the immediate premenopausal years the distribution of the MD grades is different from other age periods. Fewer cases fall into the poor prognosis M++D− grade and more into the good prognosis M++D++ grade. Furthermore, in this series, the 15-year survival figures for each grade, including the M++D− grade are better in this premenopausal age group than at any other time of life. These figures are small and, of course, not significant statistically but it is an interesting trend which is consistent and goes some way to explaining the better prognosis enjoyed by patients developing carcinoma of the breast in this age group. It does not, of course, in any way explain the increased HDR nor the apparently increased effectiveness of the HDR.

*Post-operative irradiation, staging, nodal metastases and MD grading.*—From an examination of the figures given in this section of Results it is clear that the decision to give or withhold irradiation was not based entirely on staging or the presence or absence of nodal metastases. Naturally these patients were treated by a number of surgeons and no doubt their views on treatment of breast carcinoma varied considerably. An analysis of the proportion of patients with carcinoma of the breast treated with irradiation at RMH during the years 1935–43 inclusive, shows a marked increase from 38 % in 1935 to 82 % in 1941, dropping back to 70 % in 1942 and 1943. This suggests that the popularity of the treatment was an important factor in the decision in the later years, rather than the clinical stage or presence of nodal metastases.

The figures given in Results show a trend which suggests that post-operative irradiation does not improve the prognosis in any group of cases and may, in fact, be associated with poorer survival figures.

When these figures are taken in conjunction with MD grading the possibility that irradiation may reduce the HDR and thus worsen the prognosis is suggested by the poorer survival of the irradiated cases in comparable groups. Again these figures are very small and cannot be considered significant but it is interesting that the trend should be present and should be consistent in all stages and grades.

*Tumours of the inner quadrants.*—The poorer prognosis of inner quadrant tumours is well documented. Handley and Thackray (1954) found the presence of metastases in the internal mammary nodes to be three times more frequent with inner quadrant tumours than with outer quadrant tumours. In the present series inner quadrant tumours had a 15-year survival of 25·1 % and outer quadrant tumours 34·3 %. The distribution of the MD grades was more or less the same, but in each comparable grade and stage the percentage 15-year survival was less in the inner quadrant group, although the percentage survival did improve as the HDR score rose. Thus the poorer prognosis cannot be explained by differences in MD grading.

A slightly larger proportion of patients with inner quadrant tumours received irradiation but the percentage 15-year survival was the same in the two groups. When irradiation was not given the 15-year survival in the outer quadrant tumour patients was higher by 13 %.

### GENERAL DISCUSSION

The histological appearances described in this study cannot be accepted as proof of either the existence of an immunological defence, or of its intensity, though there is now much experimental support for such an interpretation. Nevertheless, there is no doubt of the close correlation between the intensity of the histological changes described, and prognosis. Furthermore this correlation is largely independent of other factors hitherto regarded as of prognostic significance and accounts for many of the apparently paradoxical situations encountered clinically.

The relationship of the histological changes (HDR) to nodal metastasis is interesting. The tumours of high grade malignancy (M++) with low scoring

HDR (D−) were almost invariably associated with the presence of nodal metastases and in this group of cases the clinical state of the axillary nodes is a good index of prognosis. However, the presence of axillary nodal metastases is not incompatible with a high HDR score and a long survival. The long survival without recurrence of this last group of patients may perhaps be considered an argument in favour of the continuation of radical mastectomy as the operation of choice.

The different distribution of the MD grades in the various age groups and the association of a lower incidence of the poor prognosis M++D− grade tumour in the immediate premenopausal patient does help to explain the lower mortality of this group as opposed to other age groups, both older and younger.

The depressant effect of irradiation on the function of the lymphoid system and the circulating lymphocyte count has been known almost since irradiation was first used (Hektoen, 1915, 1918). If the HDR as recorded in this investigation does, in fact, reflect an immunological reaction to the tumour then the consistently poorer survival of those patients receiving post-operative irradiation may be partially the result of depression of the immunological reaction manifested by the HDR. Bond (reported in *World Medicine* Oct. 17, 1967) has stated that post-operative irradiation does not increase the survival time of patients with nodal metastases, and may, in fact, shorten the survival period of patients without nodal metastases. The figures presented in this series show a similar trend.

### SUMMARY

A method of grading breast carcinoma, based upon the histological appearance of the tumour and the host defence reaction to it, is presented. This grading is shown to correlate closely with prognosis.

The relationship of this grading to clinical staging, nodal metastasis, age, menopausal status, and to prognosis is discussed.

The possible effect of irradiation upon a host defence reaction, which is manifested by histological appearances known to be associated with immunological reactions, is discussed.

The evidence presented supports the hypothesis that the prognosis of a patient with carcinoma of the breast is dependent on the host's reaction to the tumour as well as the grade of malignancy of the tumour.

I am very grateful to Mr. M. D. Staunton F.R.C.S. who helped me with the clinical details and restaged all the cases.

I also wish to thank Miss Ruth Bell for help with the statistics, Mr. C. H. Chadwin for technical help, the Medical Arts Department for preparation of the graphs and Mrs. M. Barry for the preparation of the photographs.

### REFERENCES

Ada, G. L., Nossal, G. T. V. and Austin, C. M.—(1964) *Aust. J. exp. Biol. med. Sci.*, **42**, 331.

Andre, J. A., Schwartz, R. S., Mitus, W. J. and Damashek, W.—(1962) *Blood.*, **19**, 313.

Baillif, R. N.—(1966) *J. Reticulo-end. Soc.*, **3**, 335.

Baldwin, R. W.—(1966) *Int. J. Cancer*, **1**, 257.

Bashford, E. F., Murray, J. A. and Cramer, W.—(1908) *Rep. imp. Cancer Res. Fund.*, **3**, 315.

BERG, J. W.—(1956) *Cancer, N.Y.*, **9**, 935.
BLACK, M. M., KERPE, S. AND SPEER, F. D.—(1953) *Am. J. Path.*, **29**, 505.
BLACK, M. M., OPLER, S. R. AND SPEER, F. D.—(1954) *Surgery Gynec. Obstet.*, **98**, 725.—(1956) *Surgery Gynec. Obstet.*, **102**, 599.
BLACK, M. M. AND SPEER, F. D.—(1958) *Surgery Gynec. Obstet.*, **106**, 163.
BLOOM, H. J. G.—(1950) *Br. J. Cancer*, **4**, 259.
BLOOM, H. J. G. AND RICHARDSON, W. W.—(1957) *Br. J. Cancer*, **11**, 359.
BOND, W. H. (reported in " World Medicine " Oct. 17, 1967, p. 68).
BRODERS, A. C.—(1920) *J. Am. med. Ass.*, **74**, 656.—(1921) *Ann. Surg.*, **73**, 141.—(1922) *Ann. Surg.*, **75**, 574.
BRUNSCHWIG, A.—(1963) *Surgery, St. Louis*, **53**, 423.
de CHOLNOKY, T.—(1943) *Surgery Gynec. Obstet.*, **77**, 55.
DA FANO, C.—(1910) *Z. ImmunForsch. exp. Ther.*, **5**, 1.
DAMASHEK, W.—(1963) *Blood*, **21**, 243.
EHRICH, W. E.—(1945–6) *Ann. N.Y. Acad. Sci.*, **46**, 823.
EVANS, W. A. AND LEUCUTIA, T.—(1939) *Am. J. Roentg.*, **42**, 886.
EVERSON, T. C. AND COLE, W. H.—(1956) *Ann. Surg.*, **144**, 366.
FAGRAEUS, A.—(1946) *Nord. Med.*, **30**, 1381.—(1948) *J. Immun.*, **1**, 58.
FOOTE, F. W. AND STEWART, F. W.—(1946) *Surgery, St. Louis*, **19**, 74.
GOWANS, J. L.—(1965) *Br. med. Bull.*, **21**, 106.
GOWANS, J. L. AND MCGREGOR, D. D.—(1965) *Prog. Allergy*, **9**, 1.
GREENOUGH, R. B.—(1925) *J. Cancer Res.*, **9**, 453.
GYLLENSTEN, L.—(1954) *Acta anat.*, **22**, 82.
HADFIELD, G.—(1954) *Br. med. J.*, ii, 607.
HAMMOND, W. G., FISHER, J. C. AND ROLLEY, R. T.—(1967) *Surgery, St. Louis*, **62**, 124.
HANDLEY, R. S. AND THACKRAY, A. C.—(1954) *Br. med. J.*, i, 61.
HANNA, M. C. JR.—(1965) *Int. Archs Allergy appl. Immun.*, **26**, 230.
HARRIS, T. N. AND EHRICH, W. E.—(1945) *J. exp. Med.*, **84**, 157.
HEKTOEN, L.—(1915) *J. infect. Dis.*, **17**, 415.—(1918) *J. infect. Dis.*, **22**, 28.
KLEIN, G.—(1966) *A. Rev. Microbiol.*, **20**, 223.
LAMBERT, R. A. AND HAINES, F. M.—(1911) *J. exp. Med.*, **13**, 505.
LUMSDEN, T.—(1925) *Lancet*, i, 383.
MACARTY, W. C. AND MAHLE, A. E.—(1921) *J. Lab. clin. Med.*, **6**, 473.
MCMASTER, P. D. AND HUDACK, S. S.—(1935) *J. exp. Med.*, **61**, 801.
MCMASTER, P. D. AND KIDD, J. G.—(1937) *J. exp. Med.*, **66**, 73.
MELLORS, R. C.—(1966) *Blood.*, **27**, 871.
MELLORS, R. C., BRZOSKO, W. J. AND SONSKIN, L. S.—(1962) *Am. J. Path.*, **41**, 425.
MELLORS, R. C., NOWOSLAWSKI, A. AND KORNGOLD, L.—(1961) *Am. J. Path.*, **39**, 533.
MOORE, O. S. AND FOOTE, F. W.—(1949) *Cancer, N.Y.*, **2**, 635.
MOTTRAM, J. C. AND RUSS, S.—(1917) *Proc. R. Soc.*, **90**, 25.
MURPHY, J. B. AND MORTON, J. J.—(1915) *J. exp. Med.*, **22**, 204.
MURPHY, J. B. AND TAYLOR, H. D. T.—(1918) *J. exp. Med.*, **28**, 1.
NOHRMAN, B. A.—(1949) *Acta radiol.*, Suppl. 77.
OLD, L. F. AND BOYSE, E. A.—(1966) *Med. clins N. Am.*, **50**, 901.
OORT, J. AND TURK, J. L.—(1965) *Br. J. exp. Path.*, **46**, 147.
PATEY, D. H. AND SCARFF, R. W.—(1928) *Lancet*, i, 801.
RICHARDS, G. E.—(1948) *Br. J. Radiol.*, **21**, 109.
RICHARDSON, W. W.—(1956) *Br. J. Cancer*, **10**, 415.
SABIN, F. R.—(1939) *J. exp. Med.*, **70**, 67.
SCOTHORNE, R. J. AND MCGREGOR, I. A.—(1955) *J. Anat.*, **89**, 283.
SMITHERS, D. W. AND PAYNE, P. M.—(1962) *Acta Un. int. Cancr.*, **18**, 906.
SOUTHAM, C. M. AND MOORE, A. E.—(1958) *Ann. N.Y. Acad. Sci.*, **73**, 635.
STEWART, F. W.—(1952) *Tex. Rep. Biol. Med.*, **10**, 239.
TREVES, N. AND HOLLEB, A. E.—(1958) *Surgery Gynec. Obstet.*, **107**, 271.